# Machine learning-driven prediction model for cuproptosis-related genes in spinal cord injury: construction and experimental validation

**DOI:** 10.3389/fneur.2025.1525416

**Published:** 2025-04-23

**Authors:** Yimin Zhou, Xin Li, Zixiu Wang, Liqi Ng, Rong He, Chaozong Liu, Gang Liu, Xiao Fan, Xiaohong Mu, Yu Zhou

**Affiliations:** ^1^Department of Orthopedics, Dongzhimen Hospital of Beijing University of Chinese Medicine, Beijing, China; ^2^Postdoctoral Research Workstation, Orthopedic Hospital, Chonqqing University of Chinese Medicine, Chongqing, China; ^3^College of Pharmacy, Gannan Medical University, Ganzhou, China; ^4^Institute of Orthopaedics and Musculoskeletal Science, University College London, Royal National Orthopaedic Hospital, London, United Kingdom; ^5^College of Integrated Chinese and Western Medicine, Changchun University of Chinese Medicine, Changchun, China; ^6^Department of Orthopedics, Qingdao Municipal Hospital, Qingdao, Shandong, China; ^7^Department of Orthopedics, The First Affiliated Hospital of Chongqing Medical University, Chongqing, China

**Keywords:** spinal cord injury, cuproptosis, machine learning, predictive models, unsupervised clustering

## Abstract

**Introduction:**

Spinal cord injury (SCI) severely affects the central nervous system. Copper homeostasis is closely related to mitochondrial regulation, and cuproptosis is a novel form of cell death associated with mitochondrial metabolism. This study aimed to explore the relationship between SCI and cuproptosis and construct prediction models.

**Methods:**

Gene expression data of SCI patient samples from the GSE151371 dataset were analyzed. The differential expression and correlation of 13 cuproptosis-related genes (CRGs) between SCI and non-SCI samples were identified, and the ssGSEA algorithm was used for immunological infiltration analysis. Unsupervised clustering was performed based on differentially expressed CRGs, followed by weighted gene co-expression network analysis (WGCNA) and enrichment analysis. Three machine learning models (RF, LASSO, and SVM) were constructed to screen candidate genes, and a Nomogram model was used for verification. Animal experiments were carried out on an SCI rat model, including behavioral scoring, histological staining, electron microscopic observation, and qRT-PCR.

**Results:**

Seven CRGs showed differential expression between SCI and non-SCI samples, and there were significant differences in immune cell infiltration levels. Unsupervised clustering divided 38 SCI samples into two clusters (Cluster C1 and Cluster C2). WGCNA identified key modules related to the clusters, and enrichment analysis showed involvement in pathways such as the Ribosome and HIF-1 signaling pathway. Four candidate genes (SLC31A1, DBT, DLST, LIAS) were obtained from the machine learning models, with SLC31A1 performing best (AUC = 0.958). Animal experiments confirmed a significant decrease in the behavioral scores of rats in the SCI group, pathological changes in tissue sections, and differential expression of candidate genes in the SCI rat model.

**Discussion:**

This study revealed a close association between SCI and cuproptosis. Abnormal expression of the four candidate genes affects mitochondrial function, energy metabolism, oxidative stress, and the immune response, which is detrimental to the recovery of neurological function in SCI. However, this study has some limitations, such as unidentified SRGs, a small sample size. Future research requires more in vitro and in vivo experiments to deeply explore regulatory mechanisms and develop intervention methods.

## 1 Introduction

Spinal cord injury (SCI) is a condition in which traumatic violence to the spine causes spinal fractures and dislocations, resulting in damage to the spinal cord and cauda equina and impairment of motor function, sensory function, nerve reflexes, and sphincter function in the limbs below the plane of injury (1–3). Epidemiological studies over the past 30 years have shown that the incidence of SCI is increasing year by year, with the number of cases reaching 250,000 to 500,000 per year, causing huge losses and heavy burdens to patients, families, and society (1, 4). There are two main stages in the development of SCI: the first stage is primary injury, which is the result of mechanical external forces acting on the spinal cord, mainly in the form of damaged blood vessels, ruptured axons, ruptured nerve cell membranes, etc. (5–11). The second stage belongs to secondary injury, a period of delayed tissue destruction, including vascular dysfunction, edema, ischemia, excitotoxicity, electrolyte transfer, free radical production, inflammation, suppressed apoptotic cell death, etc. (12–14).

Mitochondria are semi-autonomous organelles that not only provide energy for cellular activities but also play a crucial role in the production of reactive oxygen species, programmed cell death, redox signaling, transmembrane transport of various ions and regulation of electrolyte homeostasis (15–18). Copper is among the indispensable heavy metals present in the human body. It is a cofactor for many enzymes in the body and has a strong ability to bind to proteins, as well as having strong redox properties, and it is involved in regulating and maintaining the homeostasis of the body’s internal environment (19). However, excess copper in the body promotes the accumulation of lipidated proteins and the destabilization of iron–sulfur proteins (Fe-S cluster proteins). This leads to proteotoxic stress and, ultimately, cell death; a new mechanism of cell death called cuproptosis is classified as programmed cell death (20–22). Previous studies have demonstrated that copper homeostasis is intricately associated with mitochondrial regulation (23). Copper primarily exists within mitochondria through cytochrome C oxidase (COX) and superoxide dismutase (SOD1), which directly bind to the lipidated components of the tricarboxylic acid cycle (TCA), causing the accumulation and dysregulation of these proteins, obstructing the TCA cycle and initiating proteotoxic stress, among other interventions in various biological processes (21, 24). After the occurrence of SCI, the homeostasis of the body’s internal environment suffered severe trauma, and the metabolism of copper ions was also thrown into disarray, which was inextricably linked to the abnormal fluctuation of copper ions in cuproptosis (22, 25). At the moment of SCI, the blood–brain barrier, a ‘fortress’ guarding the central nervous system, is breached, its integrity is impaired, and its permeability is instantly increased. The copper ion transport channel, which had been precisely regulated, was like a ‘city gate failure,’ and copper ions in the bloodstream took advantage of the chaos to pour into the spinal cord tissue in large quantities. The local concentration of copper ions soared dramatically, forming the copper overload phenomenon. At the same time, SCI-induced inflammatory response, oxidative stress and other ‘secondary disasters’ further interfere with the normal function of intracellular copper ion transport proteins so that the uptake, distribution, and excretion of copper ions in the cell have been ‘out of control’ (26–28). On the contrary, the copper death mechanism, the abnormal increase or decrease of intracellular copper ion concentration is the key factor that triggers this death procedure, while when copper ions are overloaded, the process of copper death is initiated; while when copper ions are deficient, the dysfunction of related proteins involved in copper transport, binding and metabolism will also affect the mitochondrial metabolism, antioxidant defenses, and other key physiological processes, making the cells more sensitive to other stress factors, and indirectly laying the groundwork for cuproptosis. The imbalance of copper ion metabolism after SCI is just like a ‘key’ that precisely opens the ‘door’ to cuproptosis, and the two are intertwined, together exacerbating the pathological process after SCI (28, 29).

Based on the above, we analyzed the differential expression and immune infiltration of 13 CRGs between SCI and non-SCI based on the SCI dataset. Subsequently, unsupervised clustering based on the presence of differentially expressed cuproptosis-related genes (CRGs) was performed to divide the 38 SCI samples into two clusters of molecules associated with copper death and to analyze the expression profile and immune profile between Clusters. The co-expression modules between Cluster C1 and Cluster C2 were identified by WGCNA, and the differential genes within the significant modules were selected for GO and KEGG analysis. We then constructed RF, LASSO, and SVM models based on the differentially expressed CRGs, determined the optimal prediction model and validated the model performance using the Nomogram model, and constructed receiver operating characteristic (ROC) curves and mountain range plots to show the prediction accuracy and expression of the candidate genes. To further validate the accuracy of the candidate genes, we constructed an SCI rat model, and behavioral scores were used to assess the validity of the model. Perfusion-fixed rat spinal cord tissue specimens were observed using electron microscopy, and candidate gene expression in the rat model was verified by qRT-PCR.

## 2 Methods

### 2.1 Data collection and processing

Gene expression data for SCI patient samples were acquired from the Gene Expression Omnibus (GEO) database^1^ in the GSE151371 dataset. A total of 38 SCI samples and 10 healthy controls were included in the test set. A total of 13 CRGs, namely Solute Carrier Family 31 Member 1 (SLC31A1), PDHB, PDHA1, LIPT1, Ferredoxin 1-mediated protein (FDX1), DLD, Dihydrolipoamide S-succinyltransferase (DLST), Dihydrolipoamide branched chain transacylase E2 (DBT), Lipoic acid synthase (LIAS), DLAT, GCSH, ATP7A and ATP7B were identified through previous studies by Tsvetkov et al. (21). The raw data from the GSE151371 dataset was subjected to RMA analysis and log2 transformation, and the data was normalized by executing the Arrays function in the “limma” package.

### 2.2 Differential expression and correlation identification of CRGs between SCI and control samples

The “limma” package (30) was used to identify the differential expression of CRGs between SCI and non-SCI samples, and the “pheatmap”, “reshape2”, and “ggpubr” packages were used to create heat maps and box plots. Correlations between CRGs with differential expression were plotted by performing the “corrplot” package (31).

### 2.3 Immunological infiltration analysis

Based on the single sample gene set enrichment analysis (ssGSEA) algorithm, the “limma,” “GSVA,” and “GSEABase” packages were used to assess the relative abundance of SCI samples versus non-SCI samples with 28 immune cell types (32). Gene sets for immune cell types were obtained from Charoentong’s study (33).

### 2.4 Unsupervised clustering based on differentially expressed CRGs

Based on differentially expressed CRGs and the “ConsensusClusterPlus” package, unsupervised cluster analysis was performed to distinguish between different molecular clusters of SCI samples (34). The 38 SCI samples were classified into various clusters utilizing the k-means algorithm, with 1,000 iterations. The maximum number of clusters was set at 9, and the optimal number of clusters was determined through the assessment of the cumulative distribution function (CDF) curve, consensus matrix, and consistent cluster score. Subsequently, PCA with t-distributed stochastic neighbor embedding (tSNE) was performed to assess whether these genes could effectively differentiate between SCI samples (35, 36).

### 2.5 Cluster expression profile and immune infiltration characteristics

Analysis of cluster-to-cluster expression and immunity was constructed. The ‘ggpubr’ and ‘reshape2’ packages were executed to analyze the expression of CRGs between Cluster C1 and Cluster C2. The immune infiltration of Cluster C1 and Cluster C2 was assessed based on the ssGSEA algorithm.

### 2.6 Cluster-based clustering for weighted gene co-expression network analysis (WGCNA) and enrichment analysis

WGCNA is a systematic biological method that can be used to computationally analyze correlations between genes in microarray profiles (37). The “WGCNA” package was performed to identify co-expression modules between Cluster C1 and Cluster C2. WGCNA analysis was performed on the genes with the highest variability in the top 25%, thus ensuring the quality and accuracy of the results. A weighted adjacency matrix was constructed after determining the optimal soft threshold, which was further converted to a topological overlap matrix (TOM). Minimum module size = 100, modules were obtained using a TOM dissimilarity measure (1-TOM) based on a hierarchical clustering tree algorithm. Each module was given a random color. The signature genes within the module represent the overall gene expression profile in each module. Modular significance (MS) indicates the relationship between the module and the disease, and gene significance (GS) represents the correlation between the gene and the clinical phenotype (38). Differential genes between Cluster C1 and Cluster C2 within significant modules were obtained and imported into the David Online Platform^2^ for Gene Ontology (GO) and Kyoto Encyclopedia of Genes and Genomes (KEGG) enrichment analysis (39), as a way to explore the potential biological functions of differentially expressed genes between clusters.

### 2.7 Constructing RF, LASSO, and SVM models based on differentially expressed CRGs

We obtained the key genes by constructing the Least absolute shrinkage and selection operator (LASSO), Support vector machine-recursive feature elimination (SVM-RFE), and Random forest (RF) algorithms to obtain the key genes for each of the three machine learning models (40). The “Venn” package was executed to take the intersection of the key genes of the three algorithms. The Nomogram model was built using candidate genes derived from the intersection, and its predictive capability was assessed through calibration curve and decision curve analysis (DCA) (41).

### 2.8 Validation of candidate genes

To further evaluate the diagnostic sensitivity of SCI candidate genes and column line graphs, we performed the “pROC” package to visualize the Receiver Operating Characteristic (ROC) curve underneath as well as the Area Under Curve (AUC) as a means to determine the candidate gene’s predictive accuracy. The expression of each candidate gene in SCI and non-SCI samples is also shown as a mountain range plot.

### 2.9 Animal experiments and validation

#### 2.9.1 Grouping of animals and establishment of SCI model rats

12 SD female rats were randomly and equally divided into sham and model groups according to the random number method, with 6 rats in each group. The study was in accordance with the requirements of the Ethical Committee of Beijing University of Chinese Medicine (approval No. BUCM-2021040802-2029).

After the rats were anesthetized with 2% sodium pentobarbital (0.25 mL/100 g) by intraperitoneal injection, they were fixed in a prone position on the operating table, prepared for skin preparation, and the area T9-T11 was marked along the mid-axis of the spine. Under aseptic conditions, a 2–3 cm incision was made at the marker, and the skin, fascia, and muscles were incised in turn to expose the spinous process, the vertebral plate and the transverse process, and the vertebral plate and both sides of the vertebral arch were carefully removed with a biting forceps to expose the T9-T11 dura. The rat was fixed on the spinal cord percussion table, and the percussion device (steel rod, 10 g) was dropped from a height of 5 cm onto the T10 segment. Successful modeling is indicated when the rat shows rapid retraction and shaking of the whole body, hematoma, and congestion on the spinal cord surface, and the dura mater is intact (42). The rats were given intramuscular penicillin sodium 8 × 10^4^ U/day for three consecutive days after surgery and urinated artificially twice daily until the rats recovered their urinary function. In the sham group, the rats were only sutured after biting off the vertebral plate to expose the spinal cord (without damaging the spinal cord).

#### 2.9.2 Behavioral scoring

In this experiment, the locomotor function of the rats was observed using the Basso, Beattie, and Bresnahan locomotor rating scale (BBB) (43), which is a two-person, double-blind method, and the scores were averaged. The score was 21 out of 21, with 0 being complete paralysis.

#### 2.9.3 Hematoxylin–eosin (HE) staining

The fixed spinal cord tissue was removed, along with the excess nerve roots. Then, the tissue was paraffin-embedded and 4-μm paraffin sections were made for backup. The spinal cord slices were rewarmed in an oven at 60°C for 30 min and then routinely dewaxed and rehydrated, followed by hematoxylin staining for 5 min, tap water for 30 s, hydrochloric acid staining for 1 s, tap water for 30 s, tap water for 30 min (return to blue), eosin staining for 1 s, and finally, after immersion in tap water for 30 s, after the initial judgment of the staining situation under the microscope, the slices could be air-dried and sealed.

#### 2.9.4 Nissl staining

After the prepared spinal cord sections were routinely rewarmed, dewaxed and rehydrated, the Nissl’s staining solution was added dropwise and placed in a 60°C warm box for 1 h, rinsed with ultrapure water for 3 times (5 min/times), and then stained with the color separation solution for 1-2 min, and then the slices could be air-dried and sealed when the background was suitable.

#### 2.9.5 Transmission electron microscopy (TEM) observation

The material was taken 3 days after modeling. After the rat had been anesthetized, the heart was perfused with 4% paraformaldehyde, and a spinal cord segment approximately 1 cm long was taken from the center of the injury. Samples for transmission electron microscopy were placed in pre-electron microscopy fixative and stored in a 4°C refrigerator immediately after sampling.

The perfusion-fixed spinal cord tissue specimens were finely trimmed to a size of 1x1x3 mm^3^ and then fixed for several days in pre-electron microscopic fixative, rinsed three times in PBS solution, and then soaked in post-fixative for 1.5 h; after rinsing, dehydration, embedding, sectioning and staining in that order. The tissues were then rinsed, dehydrated, embedded, sectioned, and stained, and then they were observed and photographed under an HT7700 (Hitachi, Japan) transmission electron microscope.

#### 2.9.6 Quantitative reverse transcription-polymerase chain reaction (qRT-PCR)

The differential expression of genes (DEGs) was additionally confirmed using qRT-PCR (Applied Biosystems, USA). Gene expression levels were normalized to GAPDH using the 2^−ΔΔCT^ method. The primer sequences employed are detailed in Table 1.

**Table 1:** The primers used in this study.

Gene	Forward primer	Reverse primer
DLST	TGCAGGAGCAGCCTGTAGTA	ATGACAGGAACCACGAGACC
DBT	GTCACCATCACCAGCCGTTACG	CTGCCATCCTTTCCTGAGCCAAC
LIAS	ATGGCTTTACGCTGCTGGGATG	GGCTGTGGCGGTGGCATATTC
SLC31A1	AACCACACGGACGACAACATCAC	CACAGGCATGGAATTGTAGCGAATG
GADPH	ACTCCCATTCTTCCACCTTTG	CCCTGTTGCTGTAGCCATATT

#### 2.9.7 Statistical methods

All the experimental results in this study were data obtained independently at least 3 times. GraphPad Prism8 software is used for statistical analysis and chart drawing of the results. The unpaired T-test was used to compare between the two groups.

## 3 Results

### 3.1 Differential expression and correlation of CRGs

**Figure 1:**
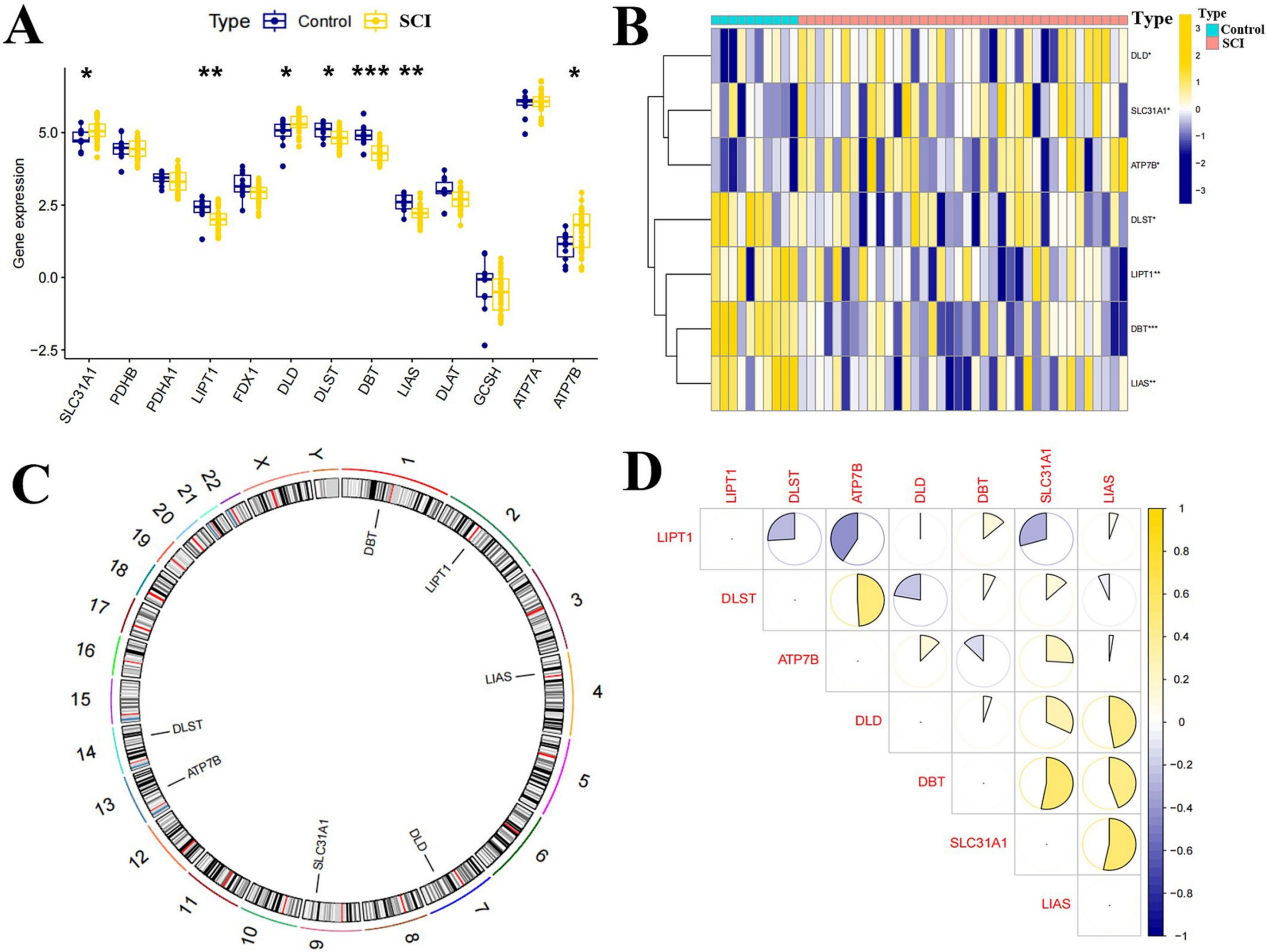
Expression and correlation analysis of CRGs with SCI. **(A)** Box plot of expression of 13 CRGs in SCI and non-SCI samples. **(B)** Heat map of 7 CRGs with differential expression. **(B)** Heat map of 7 CRGs with differential expression. **(C)** Position on chromosome of 7 CRGs with differential expression. **(D)** Correlation analysis of 7 CRGs with differential expression, yellow and blue represent positive and negative correlations respectively, area of the pie chart represents the correlation coefficient. ^***^p < 0.0001, ^**^p < 0.001, ^*^p < 0.05.

Based on the GSE151371 dataset, the expression of 13 CRGs between SCI and non-SCI samples was analyzed. The findings indicated that 7 CRGs exhibited differential expression (Figure 1A). Among them, ATP7B, DLD, and SLC31A1 showed an up-regulation trend in SCI samples, while DLST, DBT, LIAS, and LIPT1 showed a down-regulation trend (Figure 1B). The positions of the 13 CRGs on the chromosomes are shown in Figure 1C. Next, we investigated the correlation among the seven CRGs exhibiting differential expression to further explore their potential involvement in the development of SCI, explicitly focusing on their association with copper death-related genes (Figure 1D).

### 3.2 Immuno-infiltration analysis of SCI and non-SCI samples

**Figure 2:**
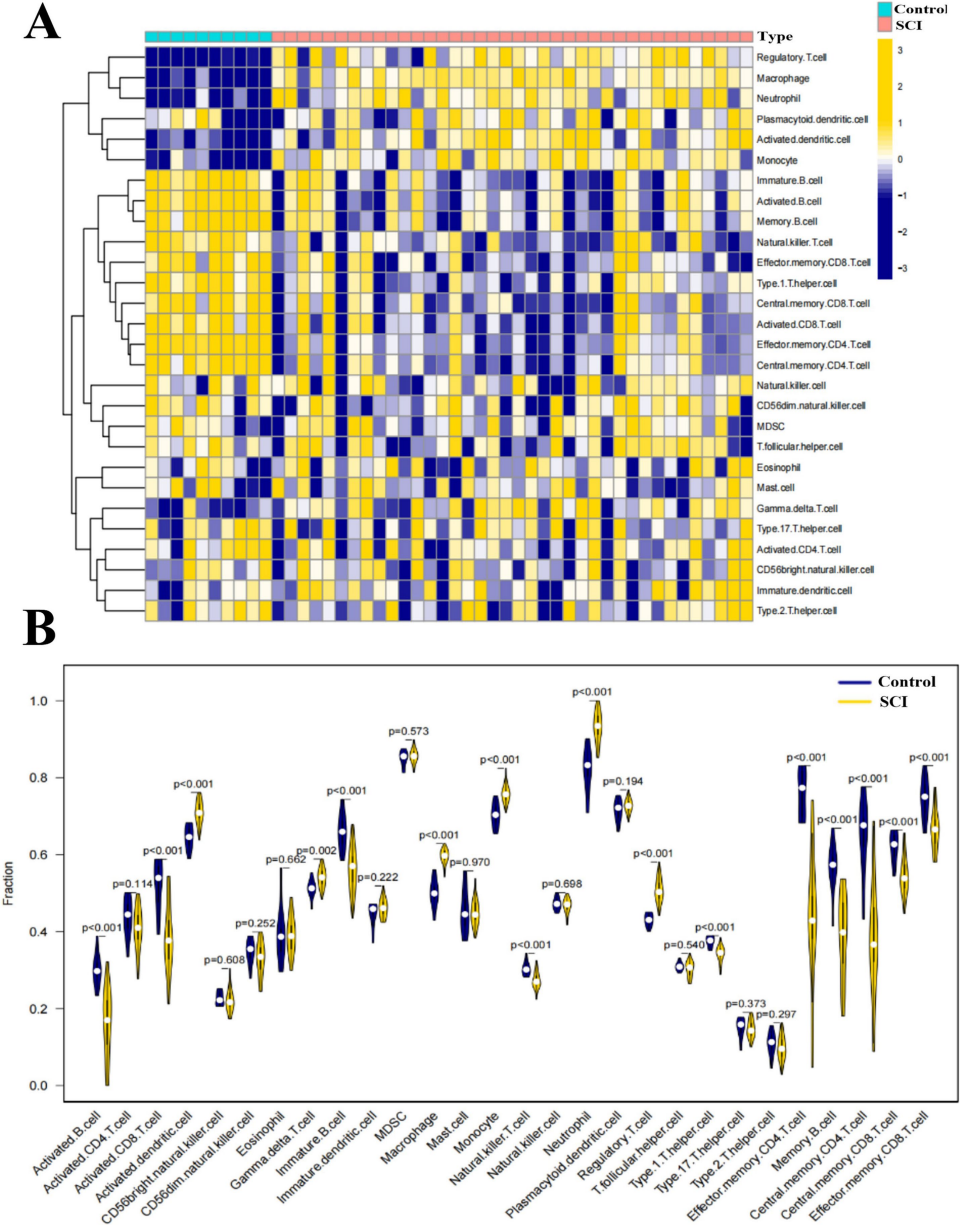
Immuno-infiltration analysis of SCI vs. non-SCI. **(A)** Results of heat map of SCI vs. non-SCI samples in 28 classes of immuno-infiltration analysis, yellow represents high expression and blue represents low expression. **(B)** Violin plot of the difference in immuno-infiltration between SCI and non-SCI samples.

The results showed that the expression levels of Activated dendritic cell, Macrophage, Neutrophil, and Regulatory T cell were significantly higher in SCI samples than in non-SCI samples (*p* < 0.001) (Figure 2A), while the expression levels of Activated B cell, Activated CD8 T cell, Immature B cell, Natural killer T cell, Type 1 T helper cell, Effector memory CD4 T cell, Memory B cell, Central memory CD4 T cell, Central memory CD8 T cell, and Effector memory CD8 T cell were significantly lower in SCI samples than in non-SCI samples (*p* < 0.001) (Figure 2B).

### 3.3 SCI unsupervised clustering analysis

**Figure 3:**
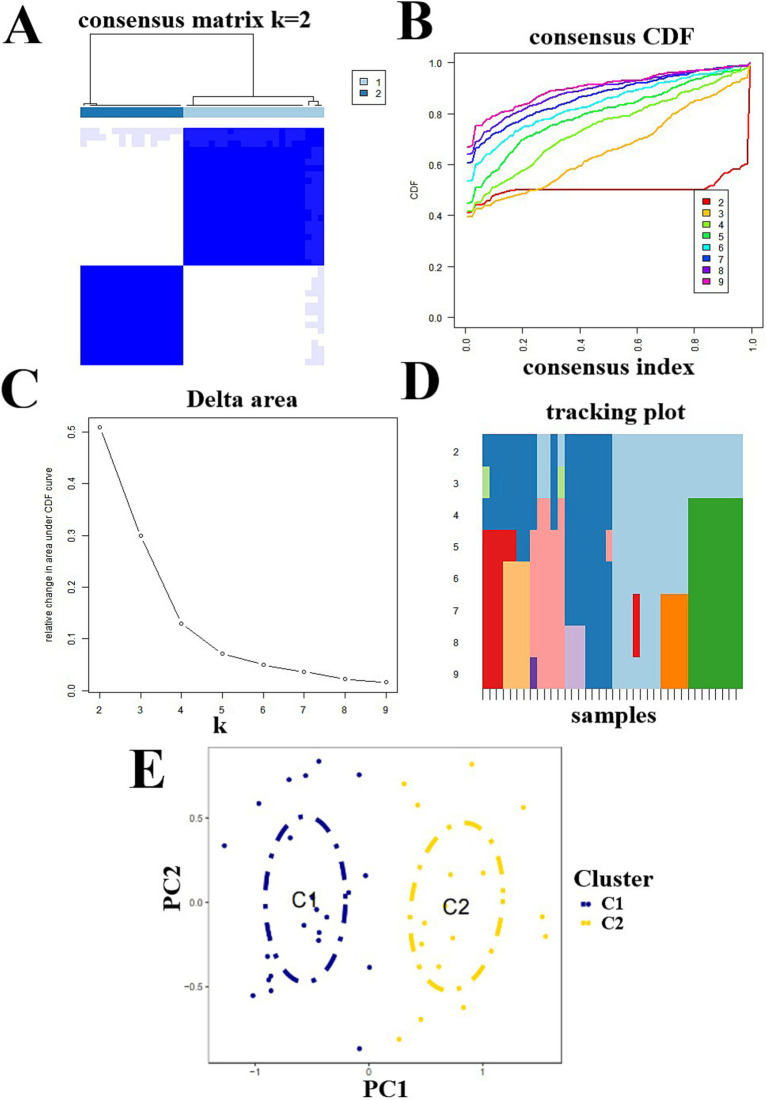
Molecular cluster identification of SCI and cuproptosis-related genes. **(A)** Consensus clustering matrix at *k* = 2. **(B–D)** Representative cumulative distribution function (CDF) curves, CDF incremental area curves, consensus clustering scores. **(E)** PCA analysis of Cluster C1 and Cluster C2 distributions.

To analyze the expression of SCI and CRGs, we identified 38 SCI samples in groups based on a consensus clustering algorithm and the expression profiles of seven CRGs. The results showed that the number of clusters was optimal when *k* = 2 (Figure 3A). The CDF values gradually increased when *k* = 2, 3, and 4 and became smaller when *k* = 4 (Figures 3B–D). We divided the 38 SCI samples into two groups: Cluster C1 (*n* = 22) and Cluster C2 (*n* = 16). PCA analysis was performed on them, and the results showed a significant difference between Cluster C1 and Cluster C2 (Figure 3E).

### 3.4 Cluster expression profile and immune infiltration analysis

**Figure 4:**
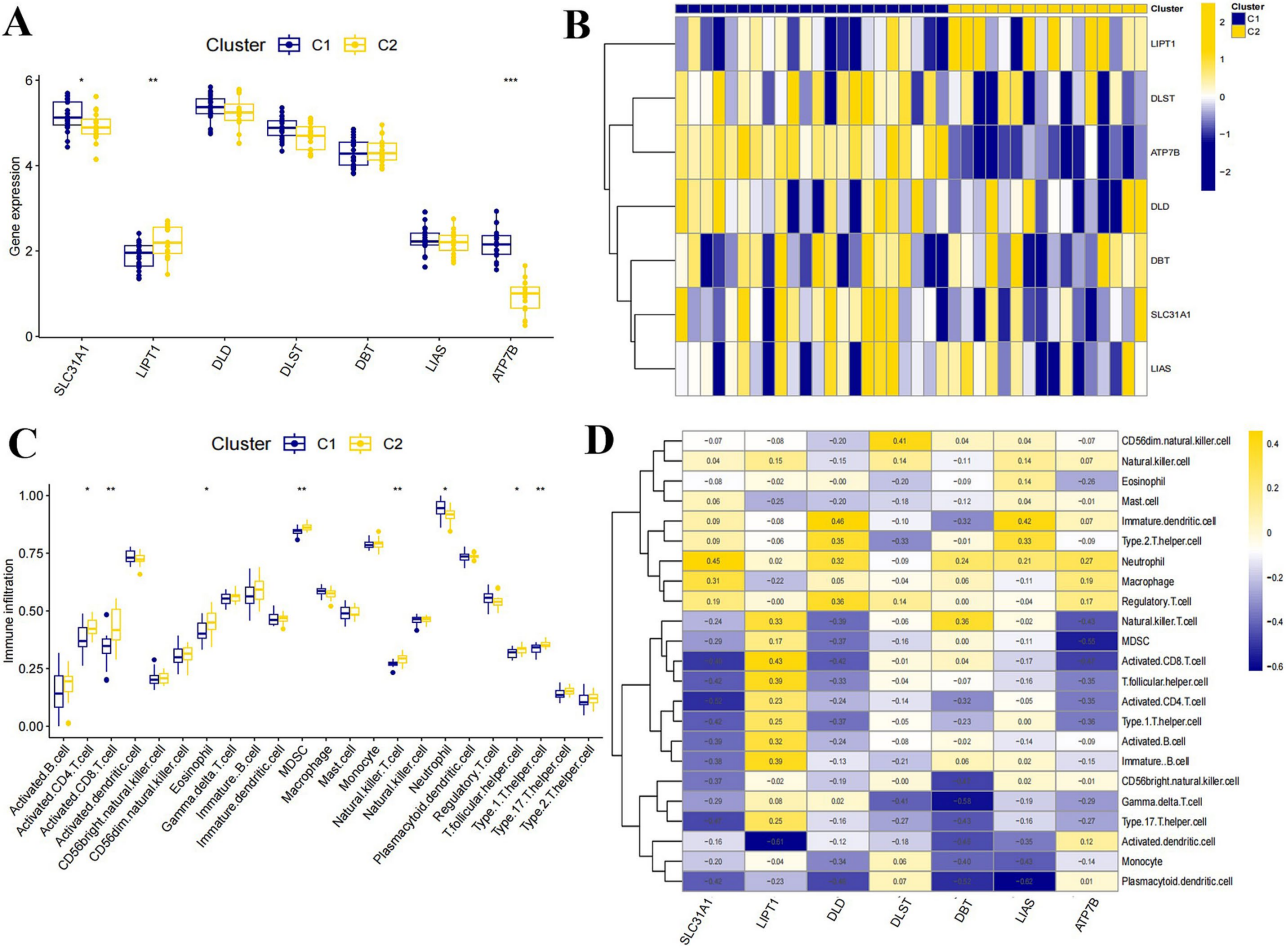
Clustered expression and immuno-infiltration analysis. **(A)** Expression of seven differentially expressed CRGs between Cluster C1 and Cluster C2. **(B)** Heat map of CRGs expression between Cluster C1 and Cluster C2. **(C)** Immunological infiltration analysis of Cluster C1 and Cluster C2. **(D)** Heat map of immunological infiltration analysis of seven differentially expressed CRGs (*p < 0.05, **p < 0.01, ***p < 0.001).

To further analyze the inter-Cluster specificity, we analyzed the expression of seven CRGs among Cluster C1 and Cluster C2 (Figure 4A). The results showed that the expression of ATP7B and SLC31A1 was higher in Cluster C1 than in Cluster C2, while the expression of LIPT1 was lower than in Cluster C2 (Figure 4B). In addition, we analyzed the immune infiltration between Cluster C1, and Cluster C2 based on the ssGSEA algorithm (Figure 4C). The findings indicated that Cluster C2 exhibited elevated levels of T cells CD8, T cells CD4 memory activated, and T cells gamma delta. In contrast, Cluster C1 displayed higher levels of T cells regulatory (Tregs), Macrophages M0, Macrophages M2, Dendritic cells activated, and increased expression of Neutrophils (Figure 4D). Based on the above, Cluster C2 appears to be more strongly associated with the progression of SCI.

### 3.5 Cluster-based screening of key modules and enrichment analysis

**Figure 5:**
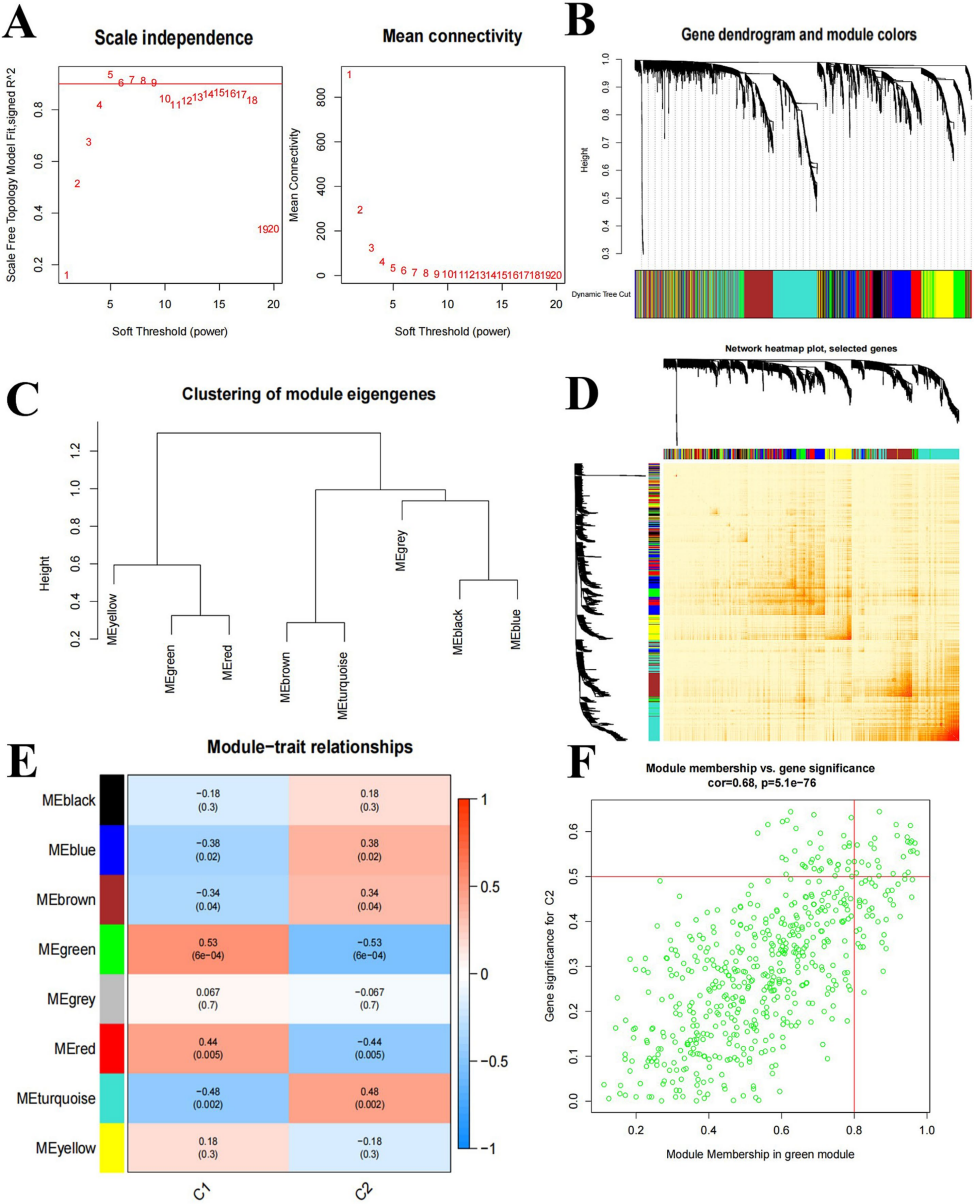
Weighted network analysis between Cluster C1 and Cluster C2. **(A)** Determination of soft threshold power. **(B)** Cluster tree dendrogram of co-expression modules; different colors indicate different co-expression modules. **(C)** Representation of clusters of module signature genes. **(D)** Representative heat map of correlations between 8 key modules. **(E)** Correlation analysis of module signature genes with clinical status. **(F)** Scatter plots between MEgreen module genes and Cluster2 significantly different genes.

We used WGCNA to analyze key modules closely related to Cluster C1 and Cluster C2. A scale-free network was constructed with *β* = 5, *R*^2^ = 0.9 (Figure 5A). The 4,375 genes were categorized into 8 significant modules, and a heat map was generated to illustrate the TOM of genes associated with each module (Figures 5B–D). Cluster C1 and Cluster C2 module relationship analysis showed that the MEgreen module (39 genes) was associated with Cluster 2 and had a high negative correlation (−0.53) with intra-module gene significance of 6e-04 (Figure 5E). MEgreen module genes were analyzed in relation to Cluster2 in Figure 5F.

**Figure 6:**
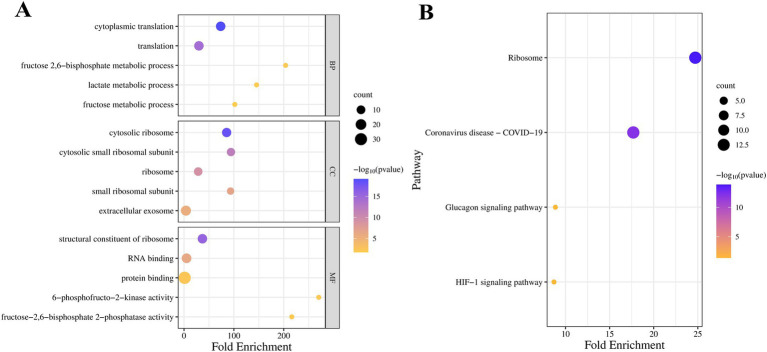
Enrichment bubble plots of three key module genes. **(A)** Bubble plots of GO enrichment analysis of three key module genes. **(B)** Bubble plots of KEGG enrichment analysis of three key module genes.

Based on Figure 5E, we selected the top 3 key modules for GO and KEGG enrichment analysis, namely MEgreen (39 genes), MEturquoise (65 genes), and MEred (12 genes), for a total of 113 genes after de-duplication of the three modules. (BP) mainly involves cytoplasmic translation, translation, fructose 2,6-bisphosphate metabolic process, etc. Cellular Components (CC) mainly involve cytosolic ribosome, cytosolic small ribosome subunit, ribosome, etc. Molecular Function (MF) mainly involves structural constituents of ribosomes, RNA binding, protein binding, etc. (Figure 6A). KEGG enrichment results showed that it was mainly involved in Ribosome, HIF-1 signaling pathway, and other related pathways (Figure 6B).

### 3.6 Identification of candidate genes based on multiple machine learning methods

**Figure 7:**
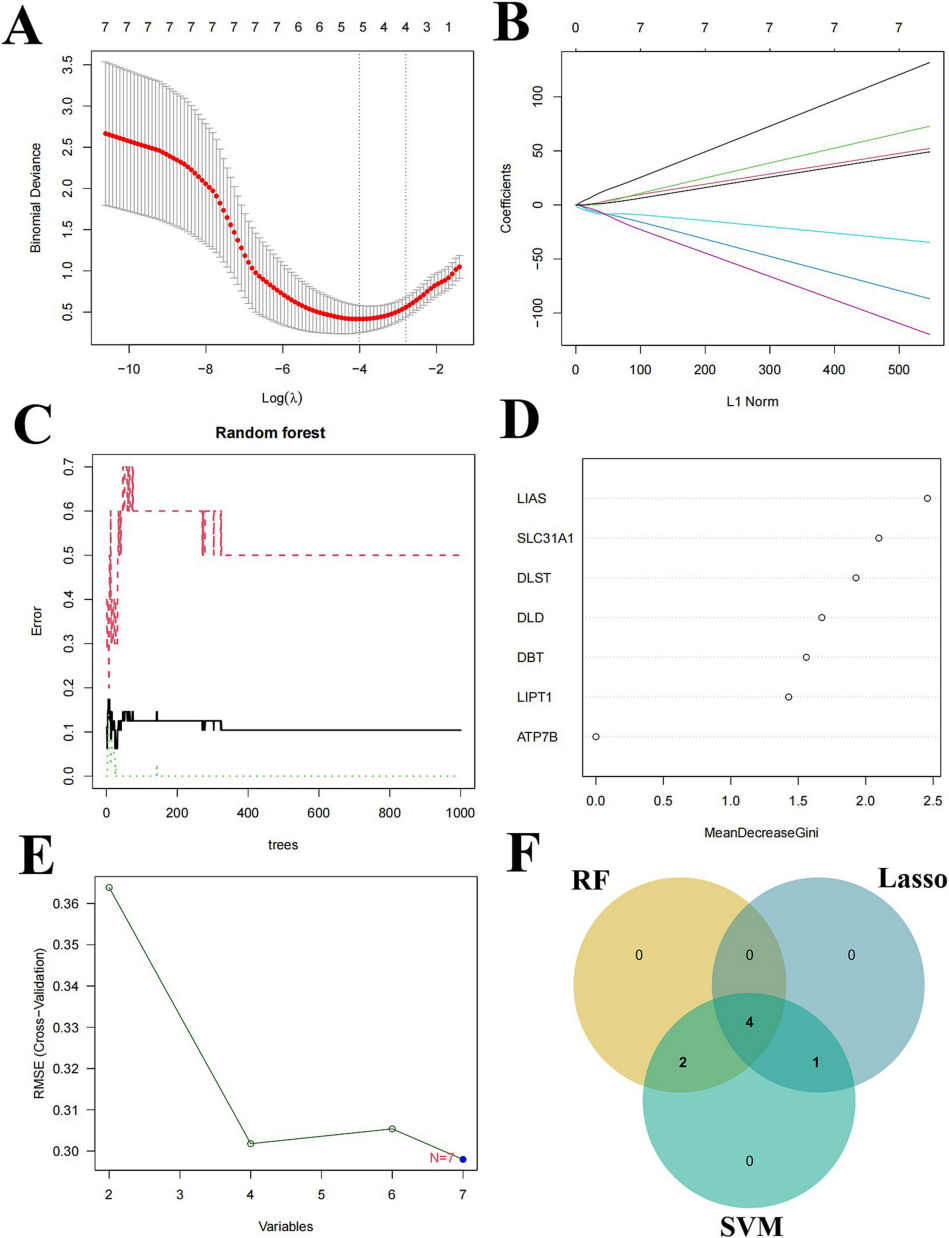
Multiple machine learning approaches to screen candidate genes. **(A,B)** LASSO analysis identifies 5 key genes. **(C,D)** RF analysis identifies 6 key genes. **(E)** SVM-RFE features identify 7 key genes. **(F)** LASSO, RF, and SVM-RFE intersection analysis yields 4 candidate genes.

To further screen CRGs for candidate genes associated with SCI, we used three machine learning methods (LASSO, RF, SVM-RFE) for feature identification based on seven CRGs with differential expression. Five key genes were obtained by the LASSO logistic regression algorithm (Figures 7A,B). Six key genes were detected by the RF algorithm (Figures 7C,D). Seven key genes were identified by SVM-RFE analysis (Figure 7E). Finally, the key genes obtained by the 3 algorithms were crossed to obtain 4 candidate genes, namely SLC31A1, DBT, DLST, and LIAS (Figure 7F).

**Figure 8:**
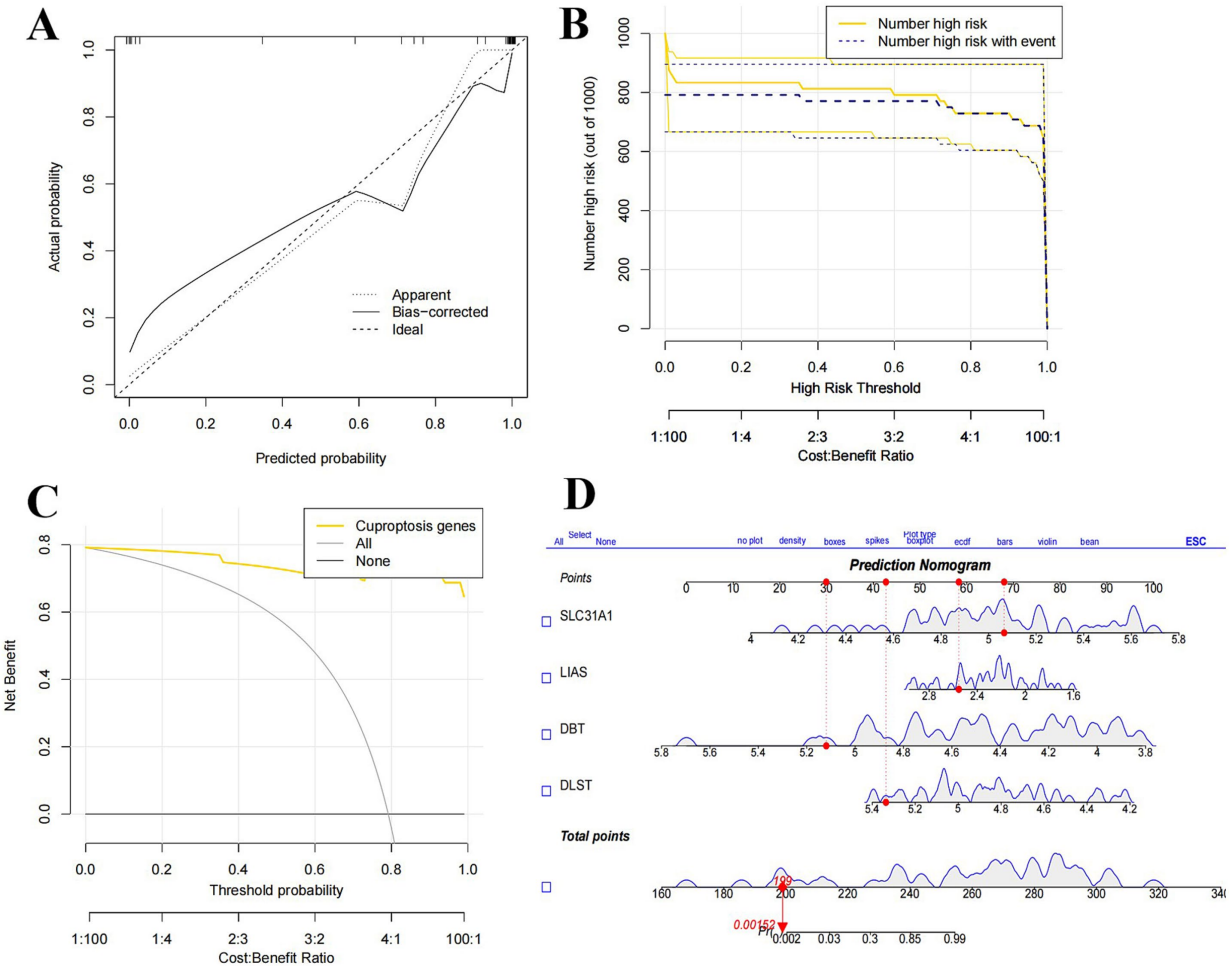
Validation analysis of candidate genes. **(A)** Construction of calibration curves. **(B)** Riskiness prediction. **(C)** Construction of DCA. **(D)** Nomogram model for predicting SCI riskiness based on 4 candidate genes.

We generated column line plots to further assess the predictive performance of the RF model (Figure 8A). The predictive performance of the generated line plot model was assessed using a combination of calibration curves and decision curve analysis. The calibration curve showed that there was a small error between the actual risk of SCI clustering and the predicted risk (Figure 8B), while the DCA results indicated that the line plot had high accuracy and could offer some reference and groundwork for clinical treatment decisions (Figures 8C,D).

### 3.7 Evaluation analysis of candidate genes

**Figure 9:**
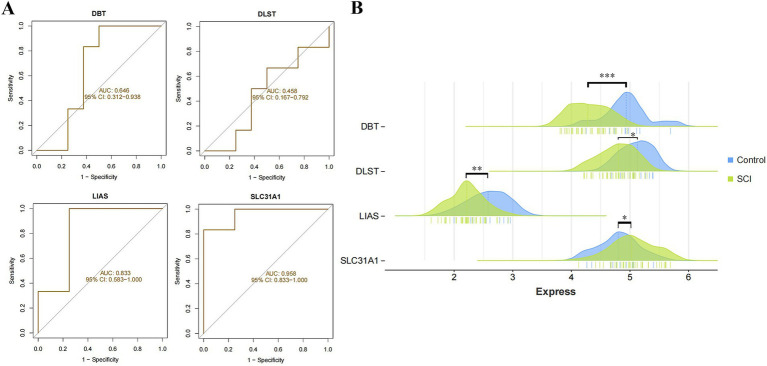
Validation and expression analysis of candidate genes. **(A)** ROC curves of the four candidate genes. **(B)** The mountain range of expression levels of the four candidate genes between SCI and non-SCI (*p < 0.05, **p < 0.01, ***p < 0.001).

We analyzed the ROC curves of the four candidate genes within the GSE151371 dataset and also contrasted their expression levels between SCI and non-SCI samples. The ROC curve results indicated that SLC31A1 exhibited the greatest relative diagnostic value (AUC = 0.958), followed by LIAS (AUC = 0.833), with a more favorable predictive effect (Figure 9A). Furthermore, the expression analysis revealed a significant increase in SLC31A1 expression levels among SCI samples compared to non-SCI samples, while the expression levels of DBT, DLST, and LIAS were all notably lower in SCI samples than non-SCI samples, all with statistical significance (Figure 9B).

### 3.8 Animal experiments and validation

#### 3.8.1 Behavioral scoring of SCI

**Figure 10:**
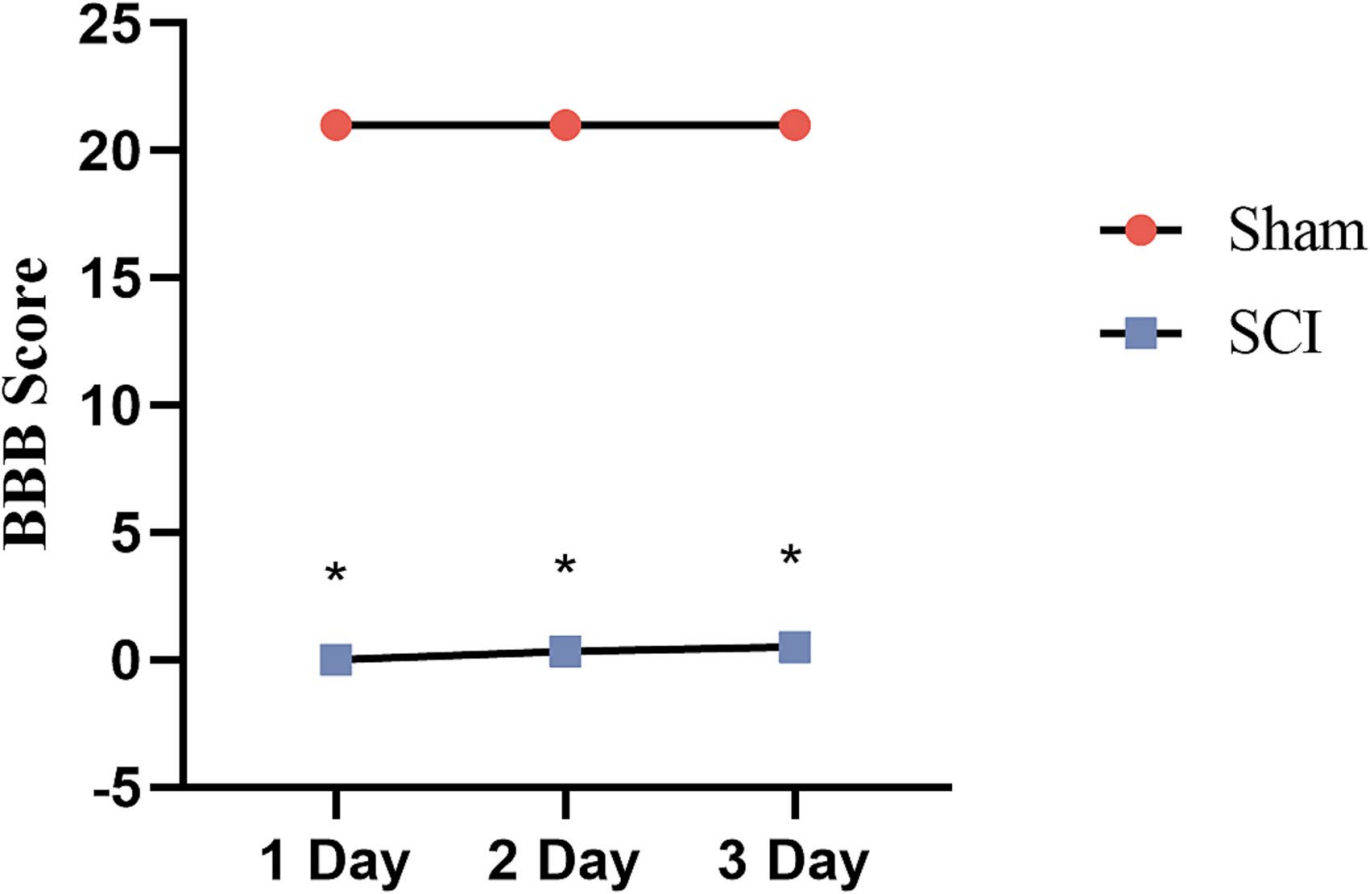
BBB scores of rats in each group. Compared with the Sham group, rats in the SCI group had significantly lower scores at the same time review (^*^p < 0.001).

The behavioral scoring of SCI was done using BBB Scale to assess the recovery of motor function in the hind limbs of rats. The assessment, with a full score of 21 points, was divided into three main sections: the movement of each joint of the rat’s lower limb, the gait and coordination function, and the precision of paw movements during lower limb activity. Our study found a significant decrease (*p* < 0.001) in the same time review score for the rats in the SCI group compared to the sham group (Figure 10), which is partly evidence of the success of our SCI model.

#### 3.8.2 Pathological tissue sections

**Figure 11:**
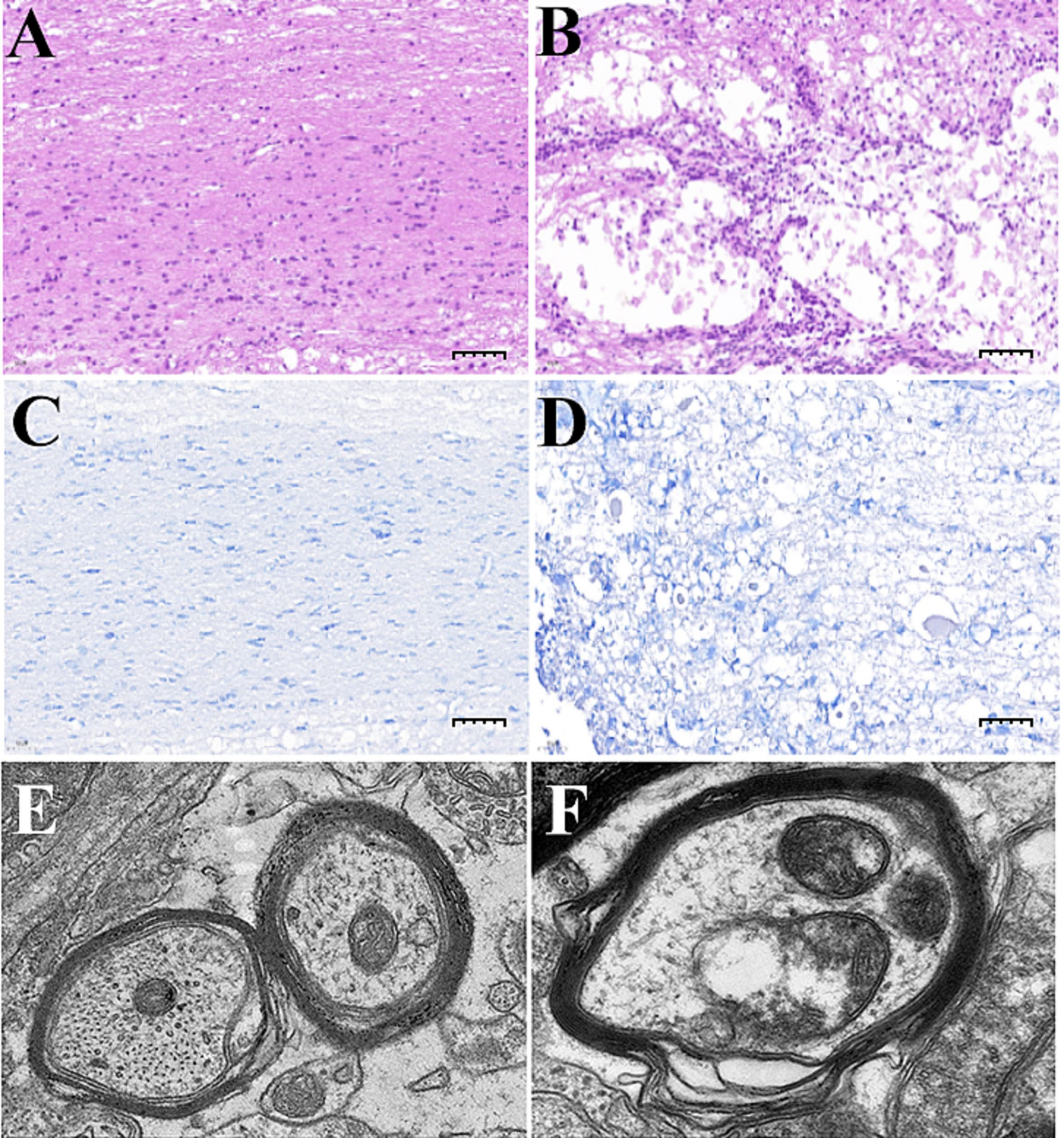
Histopathological sections of the spinal cord of rats in various groups, groups **(A,C,E)** are Sham groups and groups **(B,D,F)** are SCI. **(A,B)** HE staining of spinal cord tissue (50 μm). **(C,D)** Nissl staining of spinal cord tissue (50 μm). **(E,F)** Microstructure of myelin and neurons in the spinal cord (15000x).

In the HE-stained pathological sections, we can observe that the spinal cord tissue of the sham operation group was structurally intact, with uniform distribution of nerve cells, and no pathological changes such as vacuoles or inflammatory infiltration were seen; the spinal cord tissue of the SCI group had a large number of vacuolated changes, with neuronal atrophy and apoptosis or even disappeared, accompanied by a large number of inflammatory cells infiltration, and with structural disorders of the gray and white matter with unclear boundaries (Figures 11A,B). In the Nissl stained pathological sections, it is found that the spinal cord of the sham-operated group was structurally intact, with obvious butterfly-shaped gray matter areas and clear boundaries between the gray and white matter areas, and the neuronal cells were morphologically intact, with large nuclei and clear nucleolus, and clear intracellular tiger-spot-like Nissl bodies were seen; In the SCI group, the center of spinal cord injury was severely damaged, and stasis-like lesions were seen, as well as vacuole-like structures formed after neuronal crumpling, necrosis and liquefaction, with lighter coloring and reduced number of Nissl bodies (Figures 11C,D).

#### 3.8.3 Transmission electron microscopic observation of myelin and neuronal histomorphology

In the sham group, the spinal cord myelin sheath was regular in shape, arranged in concentric circles, with an intact structure, no rupture or loss; the laminae were intact, dense, uniform, and regularly arranged; the neuronal mitochondria were abundant, full and clearly visible, evenly distributed, microfilaments and microtubules were abundant, the neurons were oval in shape, the cell membrane was intact, the organelles were abundant, more mitochondria and rough endoplasmic reticulum were visible, and the mitochondrial cristae were not obviously broken or missing in the SCI group, the spinal cord myelin sheath was irregular in shape, disorganized in structure, with varying thickness, local curling and folding, and the laminae were loosened, twisted, folded, fused or even partially ruptured and missing. The neuronal mitochondria were swollen, and the number of mitochondria was reduced or even partially dissolved and disappeared. The mitochondrial cristae were broken and missing, and the microfilaments and microtubules were dissolved and fewer in number (Figures 11E,F).

#### 3.8.4 qRT-PCR for DLST, DBT, LIAS, SLC31A1 mRNA expression

**Figure 12:**
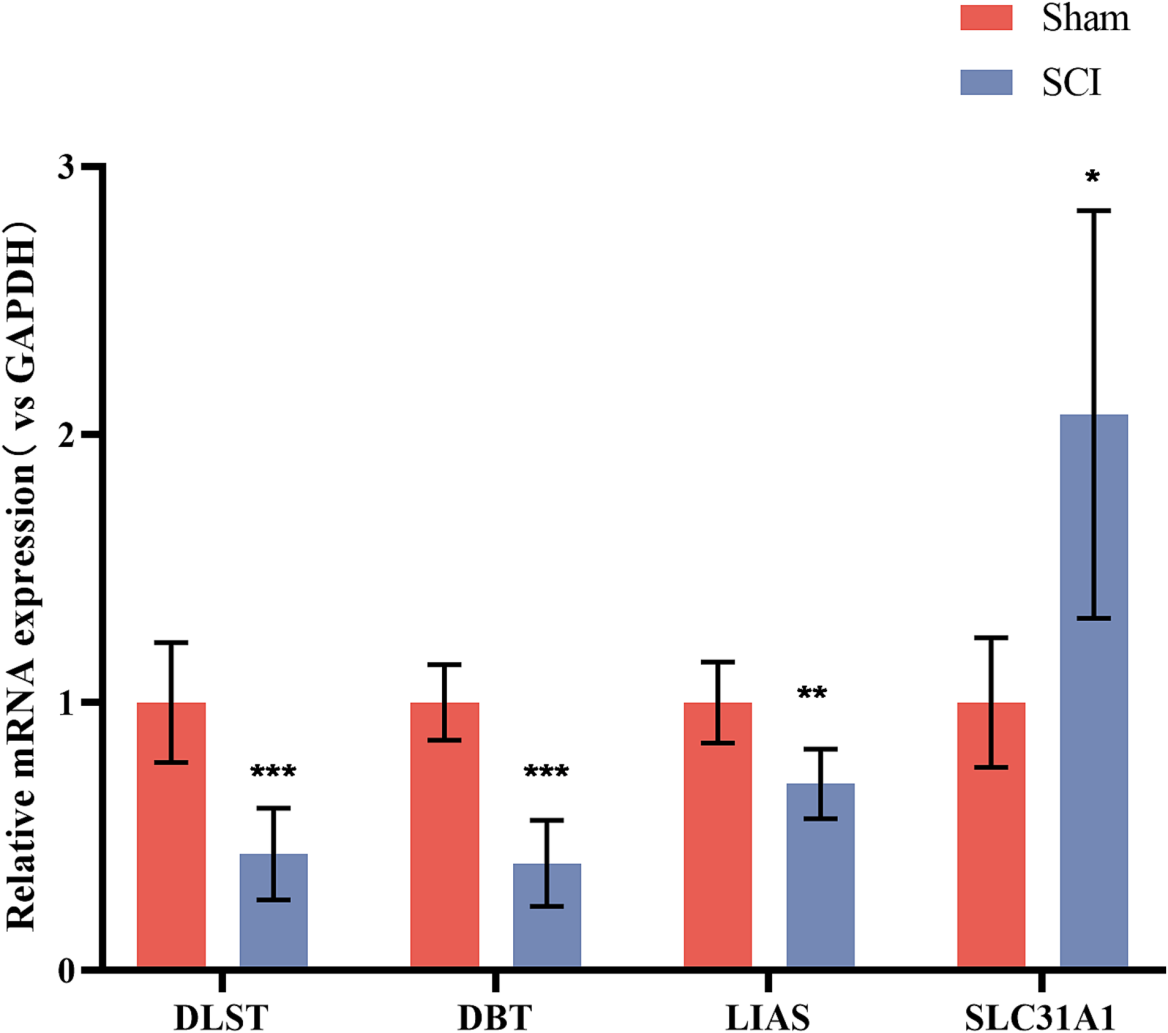
Spinal cord genes were expressed using qRT-PCR. Spinal cord genes in the SCI model and Sham group. Spinal cord expression of all 3 genes DLST, DBT, and LIAS were significantly increased in the SCI model, while SLC31A1 gene expression was significantly increased (^*^p < 0.05, ^**^p < 0.01, ^***^p < 0.001).

qRT-PCR was employed to analyze and compare the expression levels of the identified genes in the spinal cord tissues of SCI and sham groups (Figure 12). Compared with the sham group, the expression of DLST, DBT, and LIAS, 3 genes significantly decreased, whereas the expression of the SLC31A1 gene notably increased in the spinal cord tissues of the SCI rat model (*p < 0.05, **p < 0.01, ***p < 0.001).

## 4 Discussion

SCI is a devastating neurological pathology that causes major motor, sensory, and autonomic dysfunction, and the most serious damage in SCI is to the mitochondria. When mitochondrial damage leads to axonal degeneration and apoptosis (44, 45), it also causes a weakening of oxidative phosphorylation, a significant decrease in the efficiency of releasing energy through oxidation and generating ATP, disruption of Ca^2+^ homeostasis (46), and the release of cytochrome C and other pro-apoptotic proteins from mitochondria into the cytoplasm, inducing apoptosis (47); while in the secondary damage phase of SCI, vasospasm causes a lack of oxygen and energy supply to the damaged parts of the spinal cord In the secondary phase of SCI injury, vasospasm causes a lack of oxygen and energy supply to the damaged area of the spinal cord and disruption of the mitochondrial electron transport chain, resulting in damage to the mitochondria and the release of large amounts of reactive oxygen species, which in turn aggravates oxidative damage to the damaged area of the spinal cord (48, 49). It has likewise been shown that changes in mitochondrial number, localization, abundance, and substrate utilization are closely related to axonal regeneration capacity and prognosis (50–54).

Trace elements are indispensable nutrients in the animal’s body and are required for normal growth, development, and many physiological functions. Deficiencies in the balance of trace elements are closely related to a wide range of diseases and their prognosis, so maintaining normal homeostasis of all trace elements is essential for cell survival and function (55–60). Like many other metallic trace elements, copper plays an important role as one of the essential elements and as a cofactor for essential enzymes necessary for human activity (61). Under normal conditions, intracellular copper ion concentrations are generally maintained at very low levels, but when copper ions exceed the threshold, they become toxic, which in turn leads to cell death (62–64). The copper-triggered cell death that has been studied in recent years is a new model of cell death, with a specific mechanism whereby excess intracellular copper induces the accumulation of specific lipases, resulting in proteotoxic stress and, ultimately, cell death, which is linked to the mitochondrial tricarboxylic acid (TCA) cycle (21, 24).

This study marks the inaugural investigation and analysis of the varied expression of 13 CRGs between SCI patients and the healthy population. The results showed that seven CRGs exhibited distinct expression patterns between SCI patients and the healthy population, and ATP7B, DLD, and SLC31A1 were up-regulated in SCI patients, while DLST, DBT, LIAS, and LIPT1 were down-regulated. This suggests that there may be a close association between SCI and cuproptosis. Subsequently, we conducted an analysis to explore the correlation between the 7 differentially expressed CRGs, aiming to elucidate the relationship between SCI and cuproptosis. We performed unsupervised cluster analysis on 38 SCI samples based on the 7 CRGs and classified them into Cluster C1 and Cluster C2. To further explore the inter-cluster specificity, we explored the expression of the 7 CRGs among Clusters, and the results showed that ATP7B and SLC31A1 in Cluster C1 expression were higher than those of Cluster C2, while LIPT1 expression was lower than that of Cluster C2. The results of ssGSEA analysis indicated that Cluster C2 exhibited elevated levels of T cells CD8, T cells CD4 memory activated, and T cells gamma delta. Cluster C1 displayed increased expression levels in T cells regulatory (Tregs), Macrophages M0, Macrophages M2, Dendritic cells activated, and Neutrophils. Based on the above, Cluster C2 appears to be more strongly associated with the progression of SCI. Next, we performed WGCNA analysis based on Cluster C1 and Cluster C2 and selected the top 3 key modules (MEgreen, MEturquoise, MEred) genes in terms of relevance for GO and KEGG enrichment analysis, and the results showed that the above genes were mainly involved in the Ribosome, HIF-1 signaling pathway, and other pathways. Based on seven CRGs, three machine learning methods (LASSO, RF, SVM-RFE) were used for feature identification, and four candidate genes (DBT, SLC31A1, LIAS, DLST) were obtained after crossover. The ROC curve results indicated that SLC31A1 exhibited the highest diagnostic value (AUC = 0.958) relatively. According to the results of qRT-PCR experiments, the observed expression of the four candidate genes in the SCI model rats was consistent with the above analysis.

DBT constitutes a crucial element within the branched-chain α-keto acid dehydrogenase complex, an intramitochondrial enzyme complex that plays an important role in amino acid metabolism (65–67). DLST is a subunit of the alpha-ketoglutarate dehydrogenase complex, the second oxidative decarboxylation limiting enzyme of the TCA cycle, which acts mainly in the mitochondria and partly in the nucleus (68, 69). In our study, the finding that DLST expression is down-regulated in the disease would indicate that mitochondrial respiratory chain function would be impaired and energy ATP production would be reduced, failing to meet the energy requirements for repair of damaged spinal cord tissue and neuronal survival. Meanwhile, DBT and DLST are enzymes that are lipidated by copper ions, and they are involved in the metabolic complex that regulates the entry of carbon into the TCA cycle (70, 71). The DBT gene expression disorder, likewise, affects the TCA cycle, leading to disruption of the mitochondrial energy metabolism chain and impaired mitochondrial function, exacerbating the energy crisis of cells in the region of spinal cord injury, which is detrimental to the recovery of neurological function. The SLC31A1 gene, also known as copper transporter protein 1, has a high affinity for copper transport in individual cells and is a key gene for copper ion transport, which is used in the study of cancer-related therapies (64). Abnormal expression of SLC31A1 leads to insufficient or excessive levels of copper ions in mitochondria, affecting the activity of copper ion-dependent enzymes, which in turn affects the function of the mitochondrial respiratory chain, resulting in mitochondrial oxidative damage. LIAS belongs to the lipoic acid synthase family and is a highly conserved enzyme found in prokaryotes and eukaryotes. It participates in lipoic acid metabolism and lipid acylation and is an important regulator. LIAS is involved in Fe-S cluster biosynthesis, which is an important component of the mitochondrial respiratory chain complex. Abnormal expression of the LIAS gene leads to impaired synthesis of Fe-S clusters, reduced activity of the respiratory chain complex, impeded electron transfer, reduced efficiency of ATP generation, and generation of a large amount of ROS, which further damages the mitochondrial membrane and the internal structure of the mitochondrion, forming a vicious circle. FDX1 is an upstream regulator of protein lipidation and is also a direct target of copper ion carriers, which has a role in regulating protein-lipid acylation (72, 73). In previous studies, it was found that deletion of FDX1 and LIAS conferred resistance to copper-induced cell death (21), but it has also been shown that defects in neonatal LIAS are associated with severe metabolic disorders (74). Based on the above mechanism, it is not difficult to judge that the abnormal expression of the above genes also causes oxidative stress and immune response (75, 76). When oxidative stress is combined with mitochondrial injury, DAMPs will be released to activate macrophages, T cells and other immune cells, and the abnormal expression of the genes will indirectly regulate the expression of chemokines and cytokines of immune cells by influencing the function of mitochondria and oxidative stress, and affect the infiltration of immune cells into the site of SCI (77, 78). Normal immune cell function depends on mitochondrial energy supply and redox homeostasis, and mitochondrial damage and oxidative stress caused by genetic abnormalities affect immune cell activity. For example, macrophage polarization is disturbed, which prevents effective immunomodulation, and T-cell activation and proliferation are inhibited, affecting the body’s immune repair ability. Meanwhile, oxidative stress induces the release of inflammatory factors such as TNF-α and IL-1β, etc. Oxidative stress caused by abnormal gene expression promotes the expression and release of inflammatory factors by activating inflammatory signaling pathways such as NF-κB, which further recruits immune cells and exacerbates the inflammatory response, and the excessive inflammatory response will also lead to the dysfunction of the immune cells, resulting in a vicious circle and exacerbating the pathological process of SCI (79–81).

In summary, the four CRG genes, namely DLST, DBT, LIAS, and SLC31A1, obtained from this study based on the crossover of the three models of LASSO, RF, and SVM-RFE had satisfactory results in assessing the immune infiltration and subtype and pathology of SCI.

However, this study still has shortcomings. First, this study obtained a certain number of SRGs from the CellAge database, but more SRGs currently exist that have not been identified. Secondly, the sample size of the three training sets in this study was small, although we compensated by choosing external validation and experimental validation to confirm the candidate genes obtained in this study. Thirdly, although the four candidate genes eventually obtained in this study are closely associated with cuproptosis and SCI, validation by qRT-PCR alone is not sufficient, and it is expected that more *in vitro* and *in vivo* studies will be conducted in subsequent research work to confirm the above findings. Future research should focus on exploring the regulatory mechanisms of the candidate genes and developing effective interventions. Multidisciplinary cooperation and big data analysis are expected to provide a solid theoretical foundation and practical guidance for addressing the clinical challenges of related diseases.

## Data Availability

The original contributions presented in the study are included in the article/Supplementary material, further inquiries can be directed to the corresponding authors.

## Footnotes

^1^ http://www.ncbi.nih.gov/geo

^2^ https://david.ncifcrf.gov/home.jsp
