# Supplementary figures and images for: Machine learning-driven prediction model for cuproptosis-related genes in spinal cord injury: construction and experimental validation
Source: Front Neurol. 2025 Apr 23;16:1525416. doi: 10.3389/fneur.2025.1525416 (PMC12057486; doi:10.3389/fneur.2025.1525416)

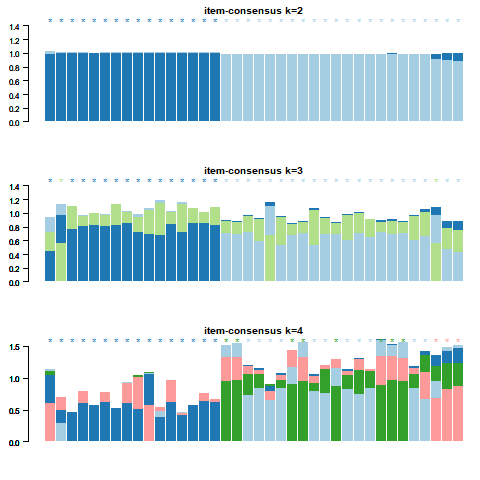

Supplement: Supplementary file 1 [file Data_Sheet_1.zip › data 1/fig.3/fig.3A-D.cluster/consensusScore/icl001.png]

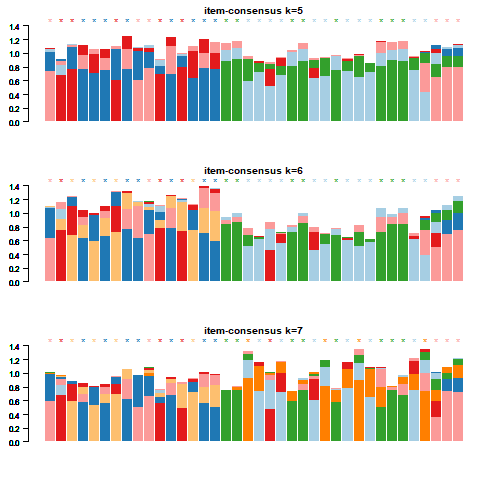

Supplement: Supplementary file 1 [file Data_Sheet_1.zip › data 1/fig.3/fig.3A-D.cluster/consensusScore/icl002.png]

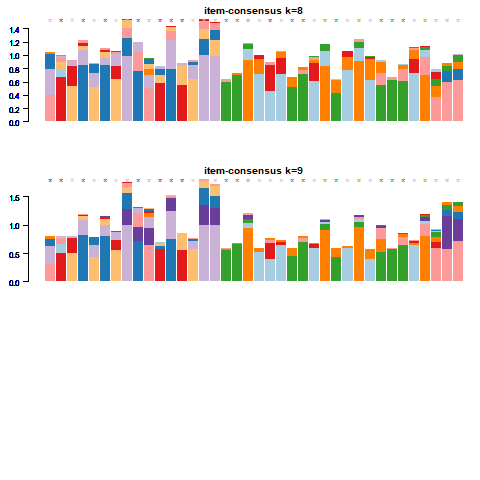

Supplement: Supplementary file 1 [file Data_Sheet_1.zip › data 1/fig.3/fig.3A-D.cluster/consensusScore/icl003.png]

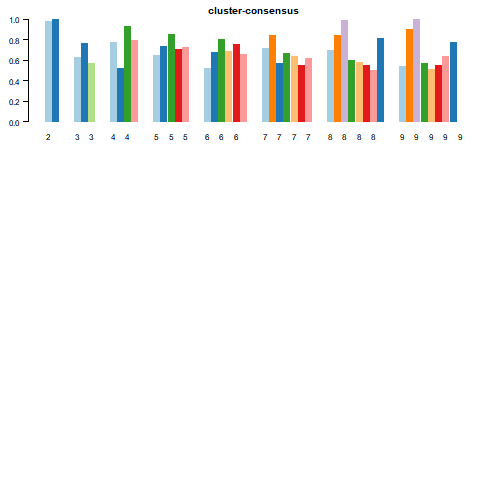

Supplement: Supplementary file 1 [file Data_Sheet_1.zip › data 1/fig.3/fig.3A-D.cluster/consensusScore/icl004.png]

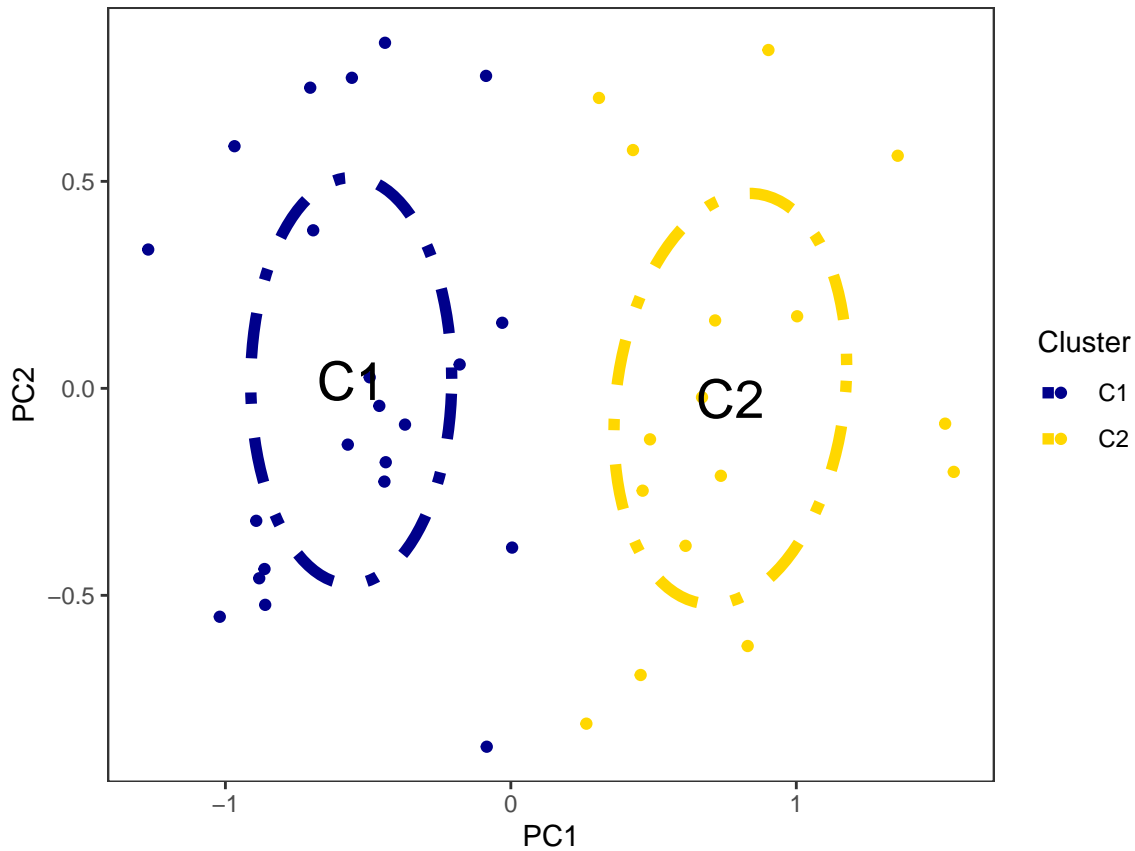

Supplement: Supplementary file 1 [file Data_Sheet_1.zip › data 1/fig.3/fig.3E.PCA/PCA.pdf]

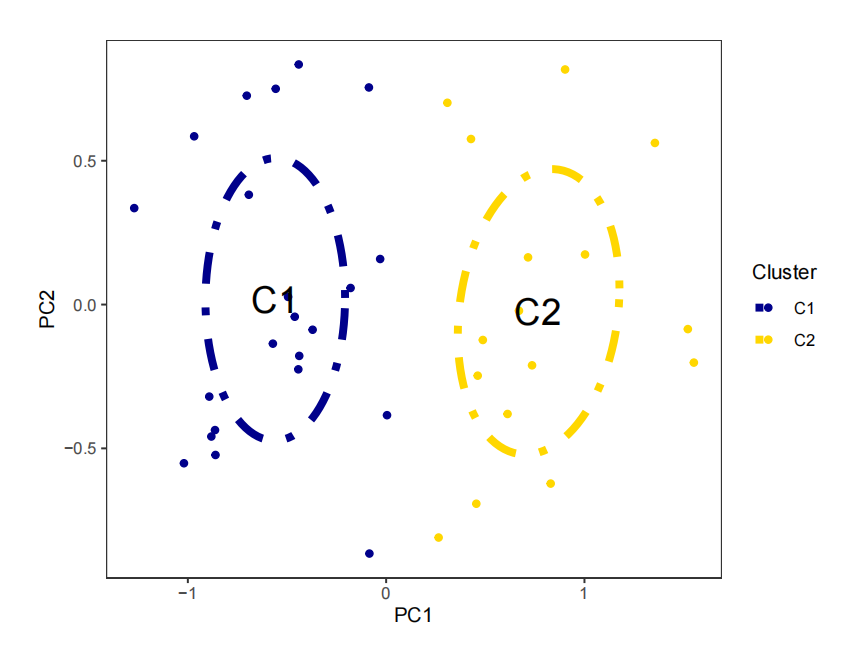

Supplement: Supplementary file 1 [file Data_Sheet_1.zip › data 1/fig.3/fig.3E.PCA/PCA_00.tif]

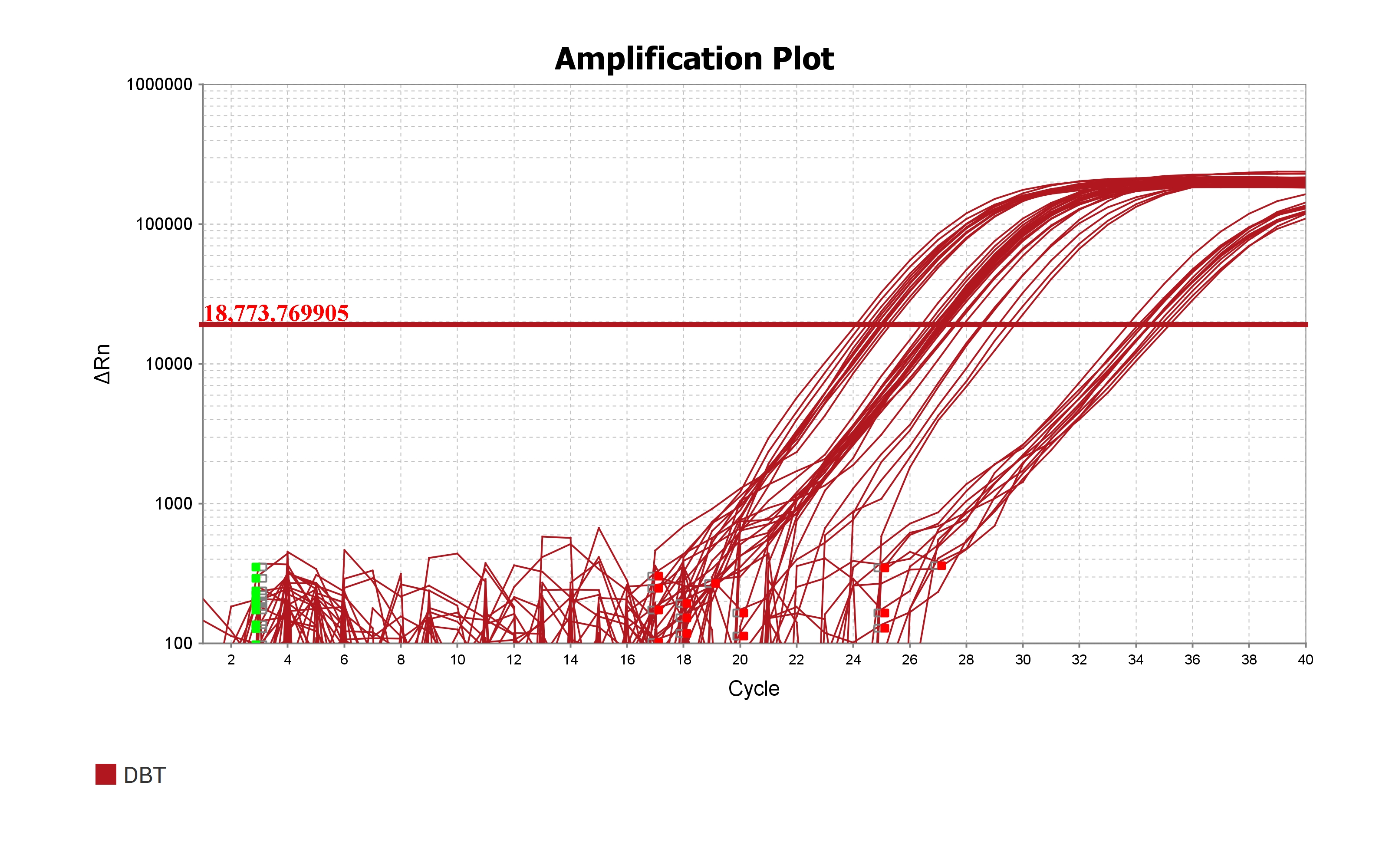

Supplement: Supplementary file 2 [file Data_Sheet_2.zip › data 2/DBT-Amplification Plot.jpg]

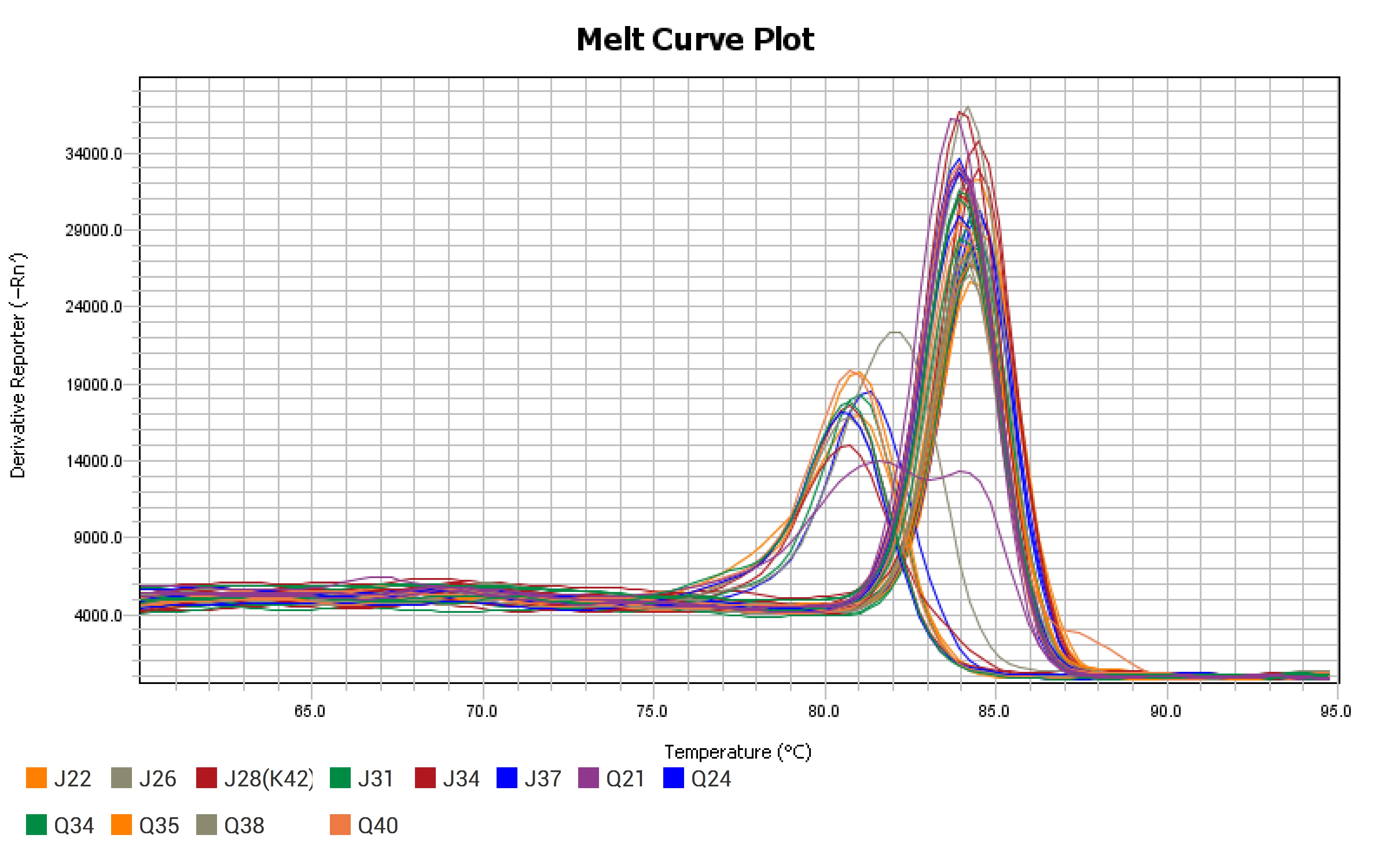

Supplement: Supplementary file 2 [file Data_Sheet_2.zip › data 2/DBT-Melt Curve Plot.jpg]

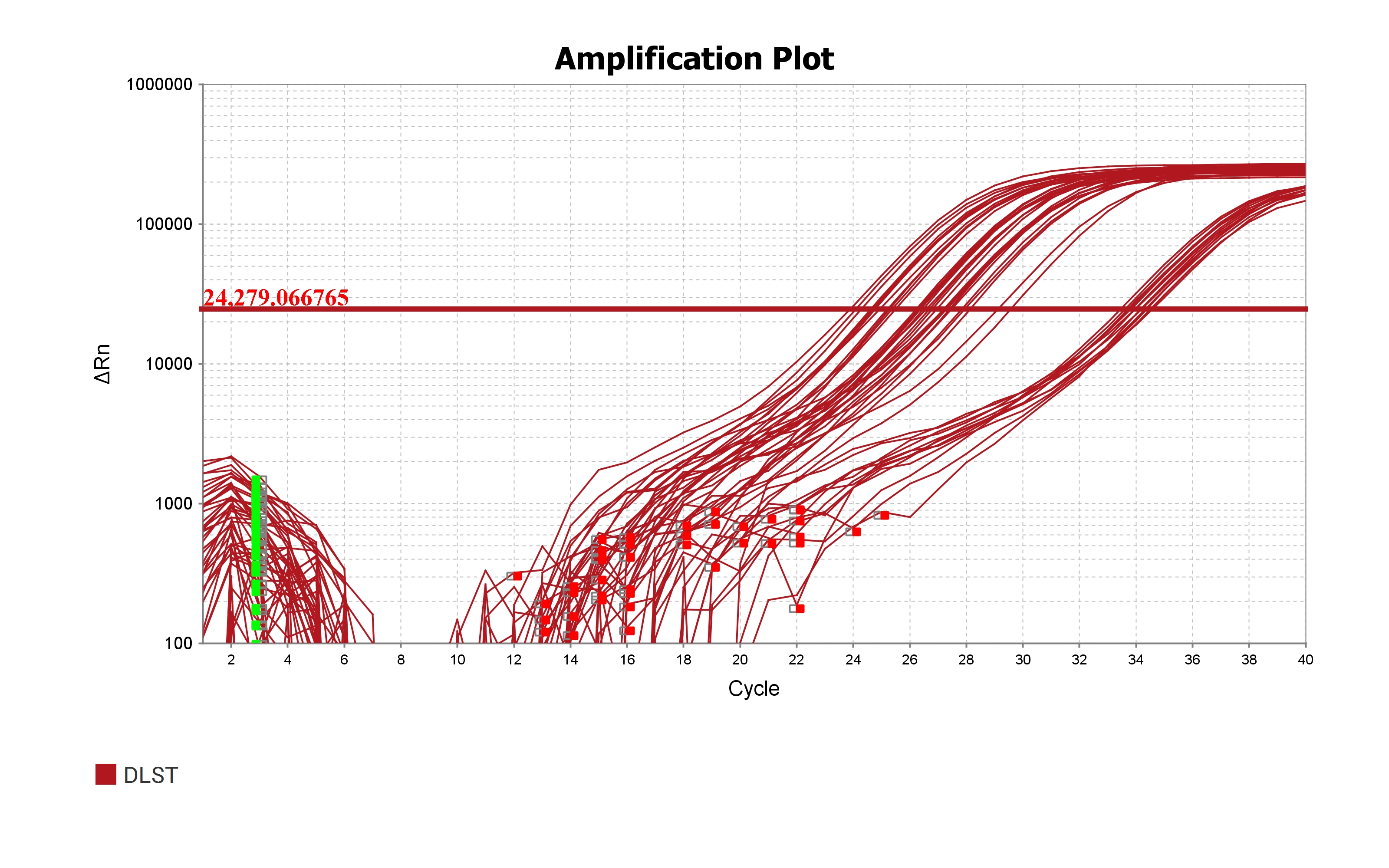

Supplement: Supplementary file 2 [file Data_Sheet_2.zip › data 2/DLST-Amplification Plot.jpg]

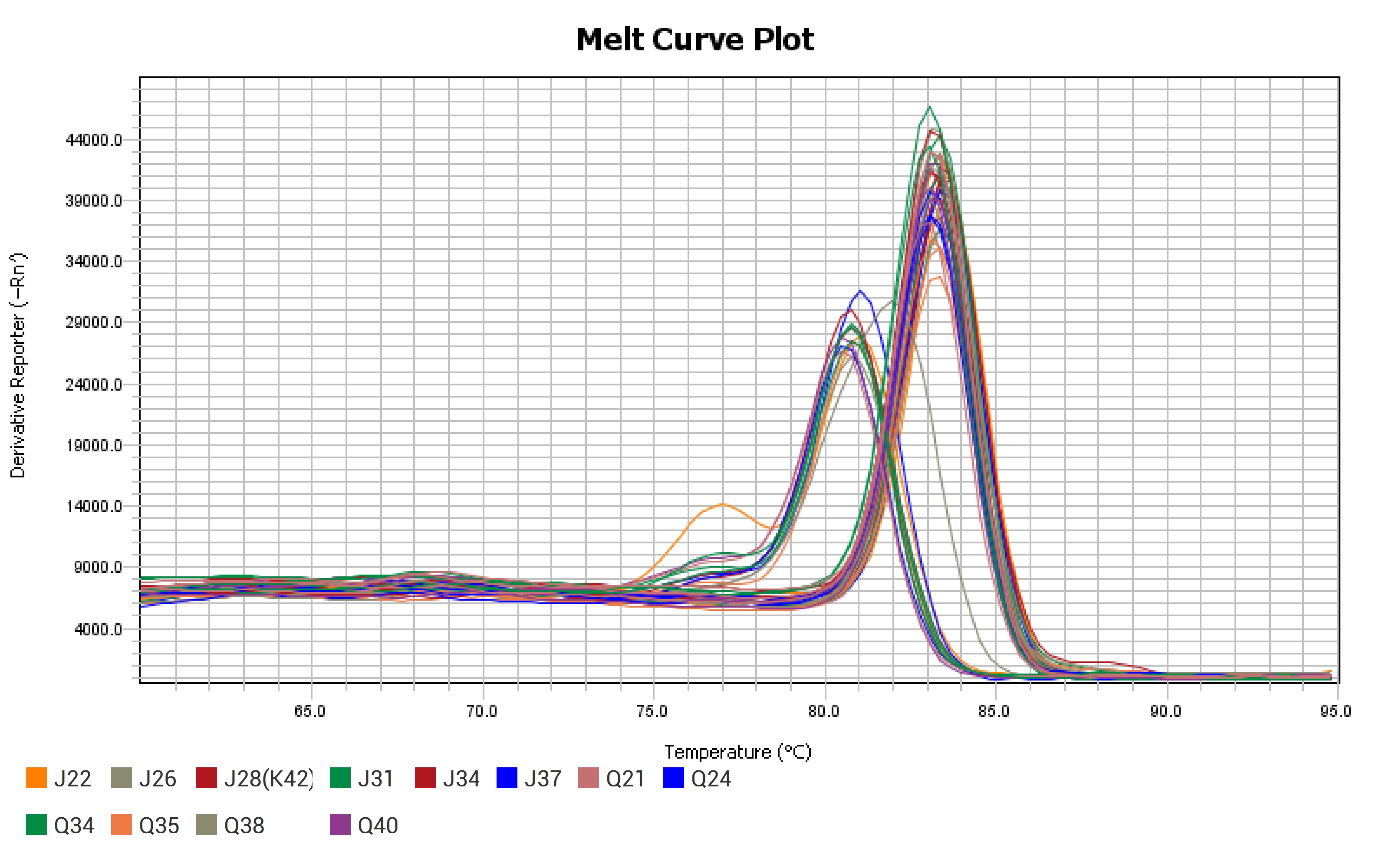

Supplement: Supplementary file 2 [file Data_Sheet_2.zip › data 2/DLST-Melt Curve Plot.jpg]

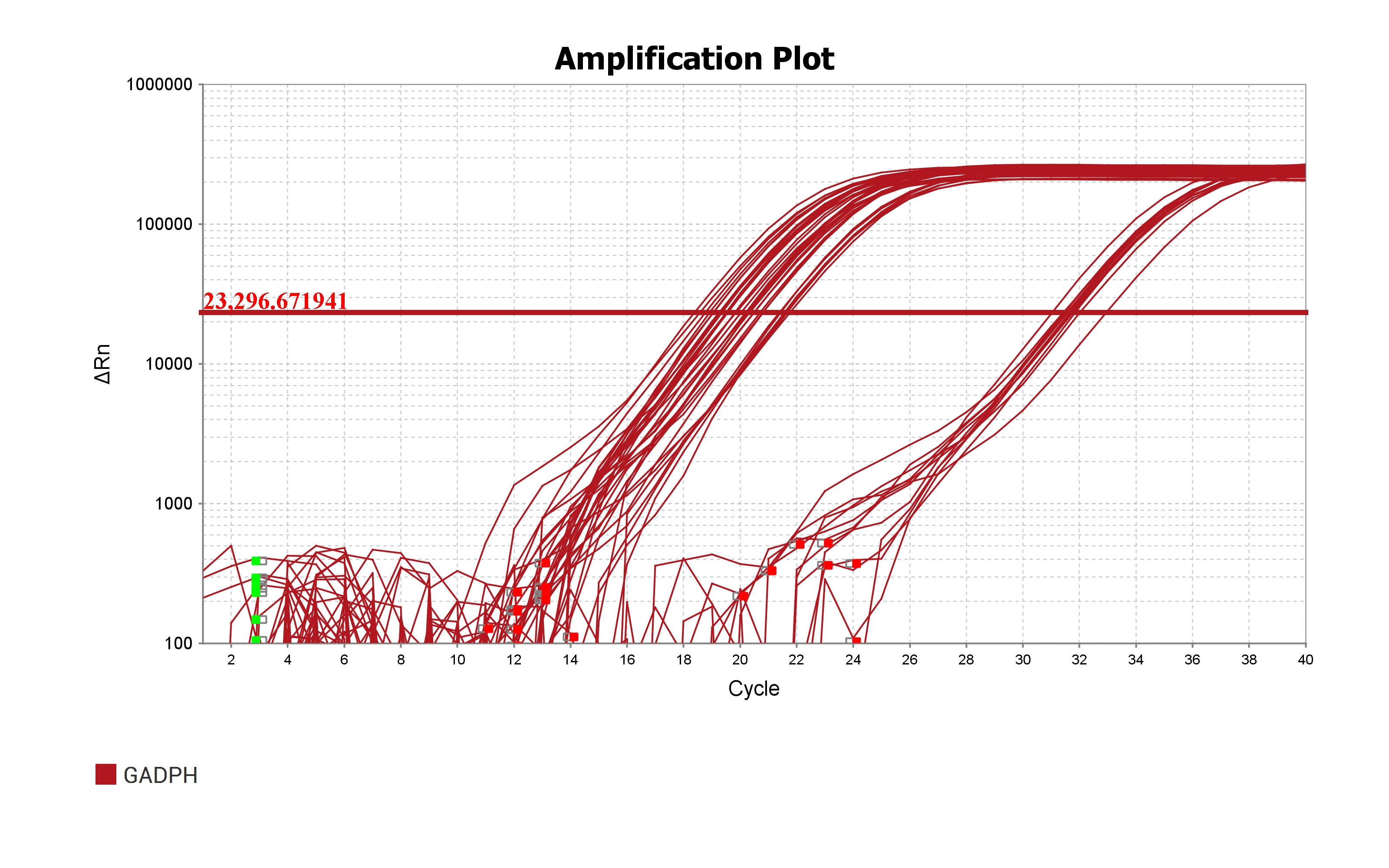

Supplement: Supplementary file 2 [file Data_Sheet_2.zip › data 2/GADPH-Amplification Plot.jpg]

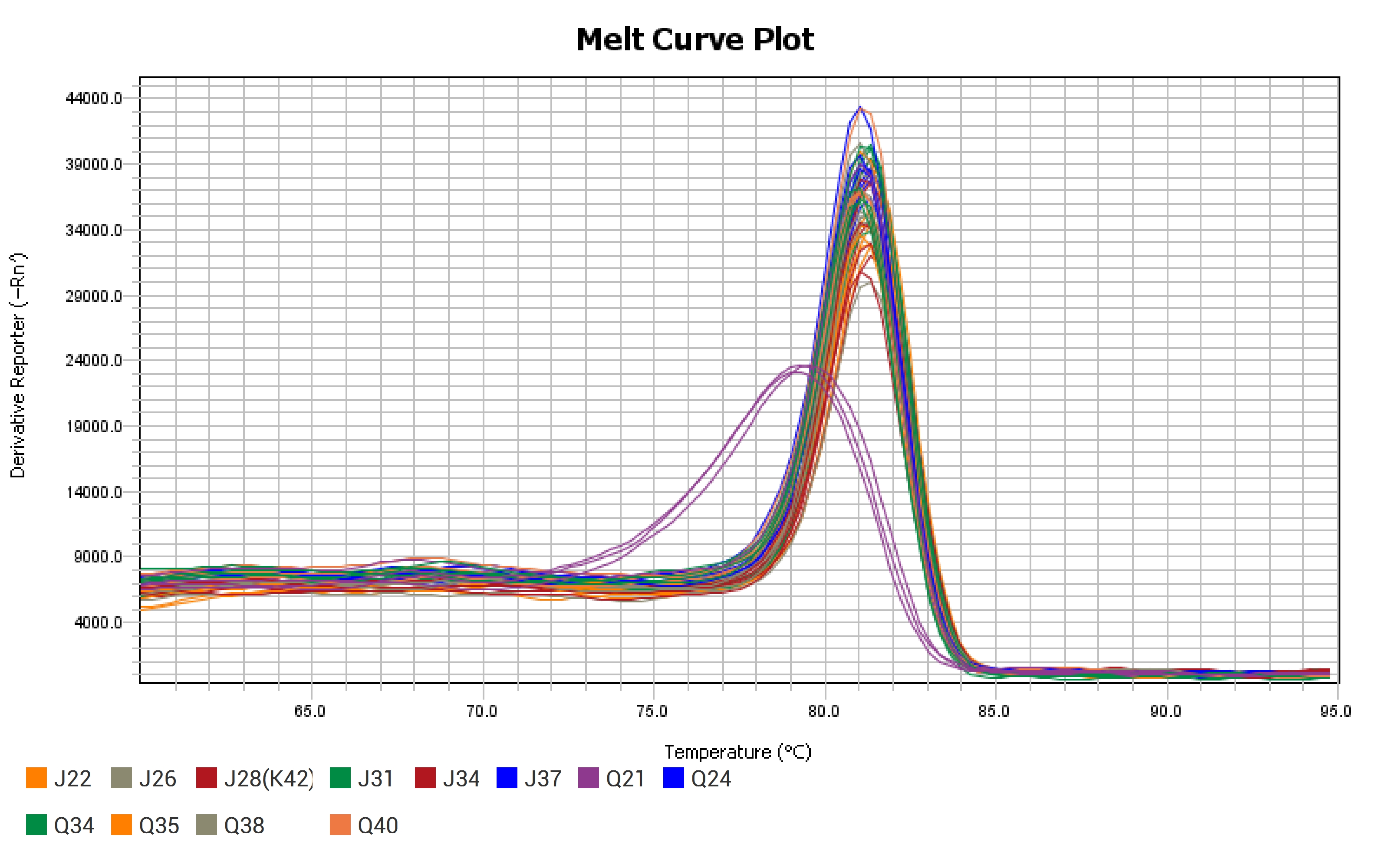

Supplement: Supplementary file 2 [file Data_Sheet_2.zip › data 2/GADPH-Melt Curve Plot.jpg]

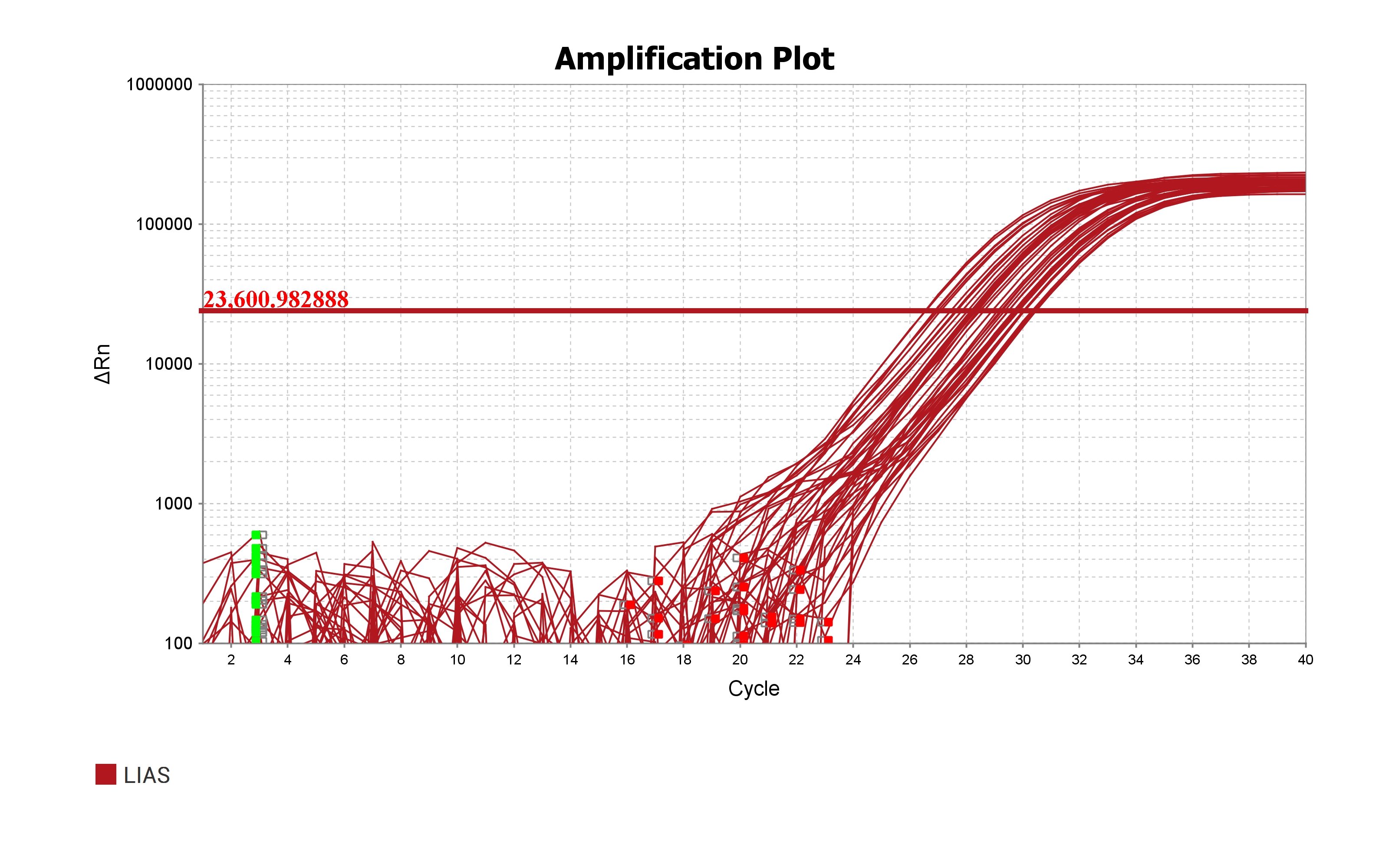

Supplement: Supplementary file 2 [file Data_Sheet_2.zip › data 2/LTAS-Amplification Plot.jpg]

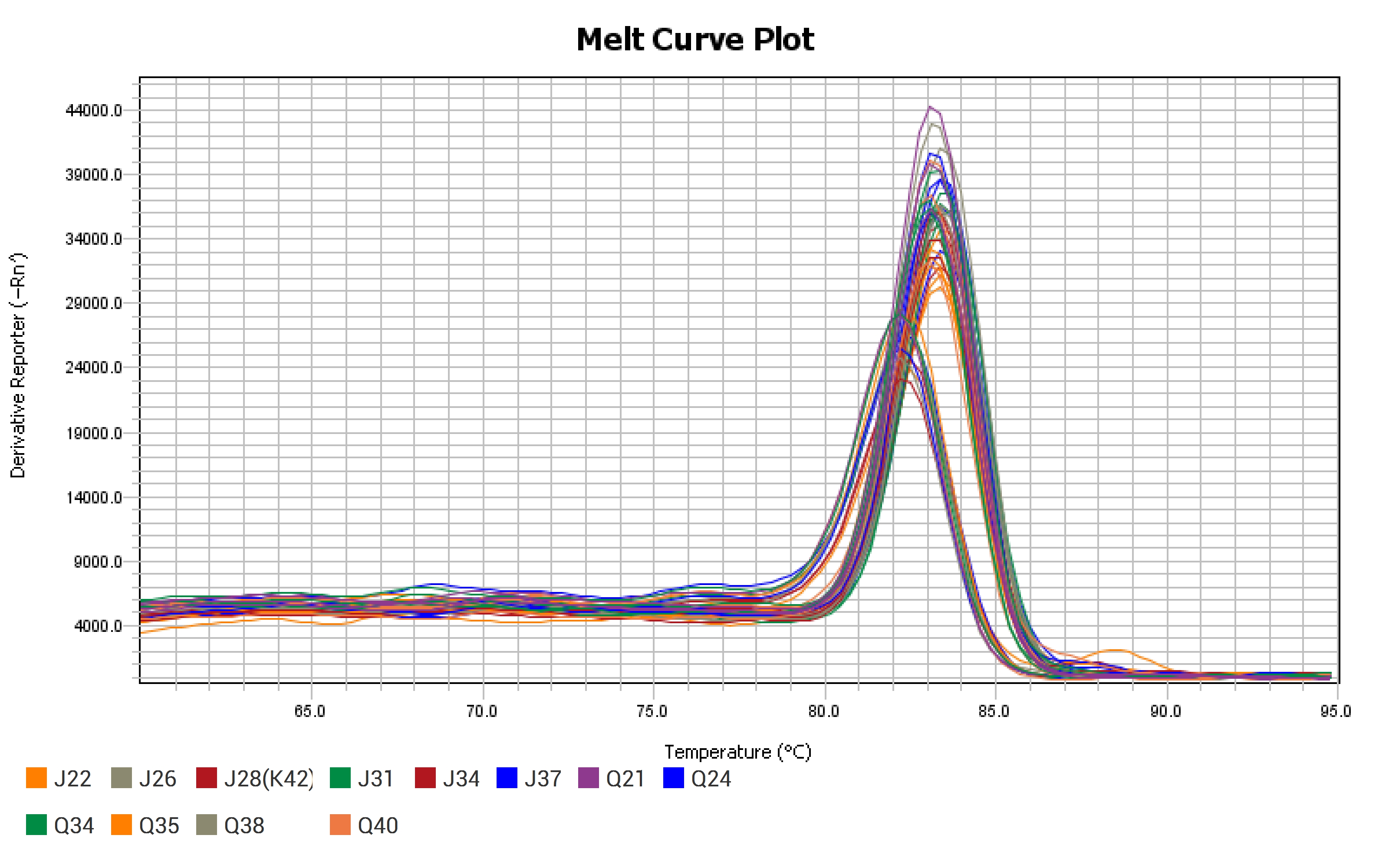

Supplement: Supplementary file 2 [file Data_Sheet_2.zip › data 2/LTAS-Melt Curve Plot.jpg]

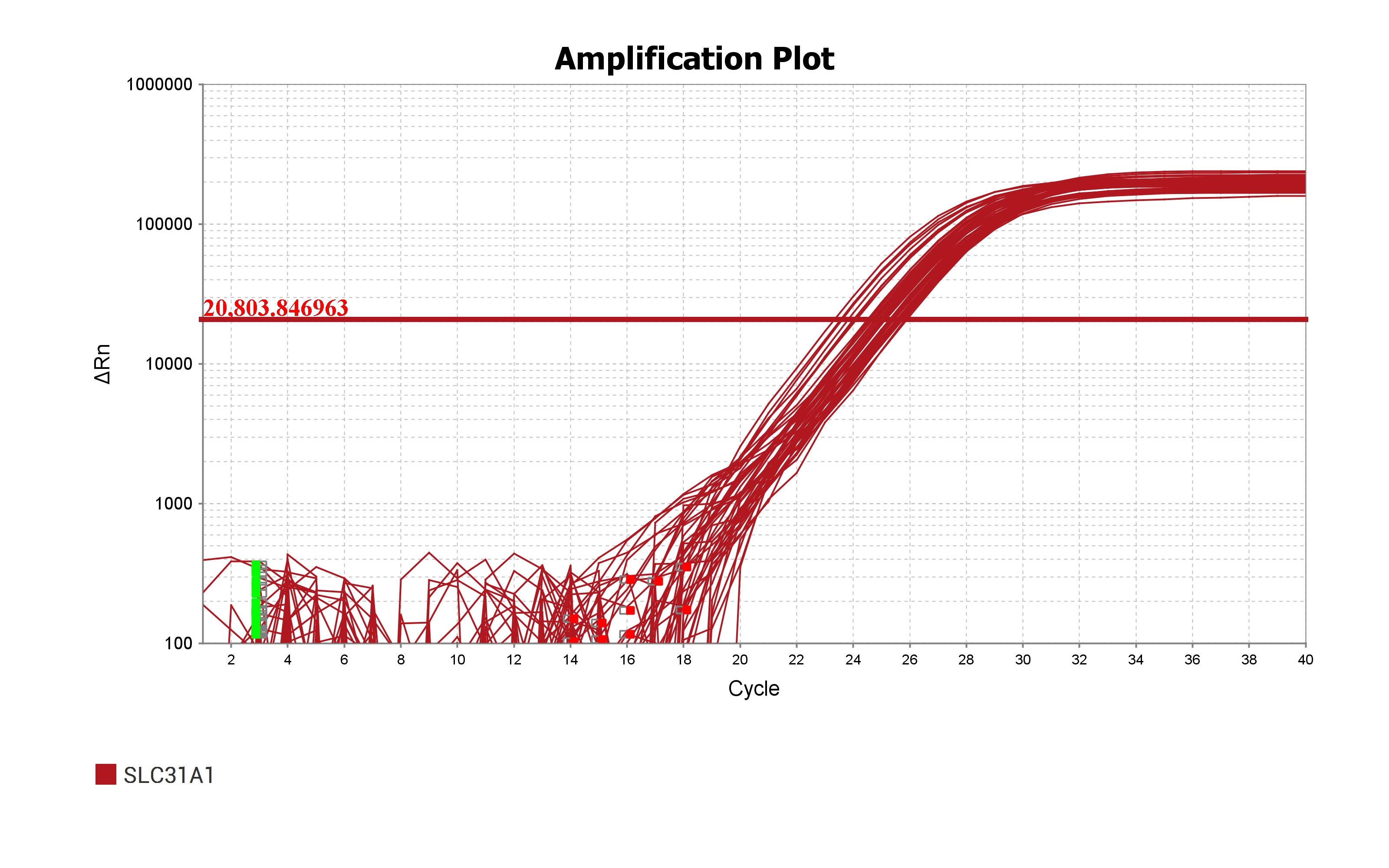

Supplement: Supplementary file 2 [file Data_Sheet_2.zip › data 2/SLC31A1-Amplification Plot.jpg]

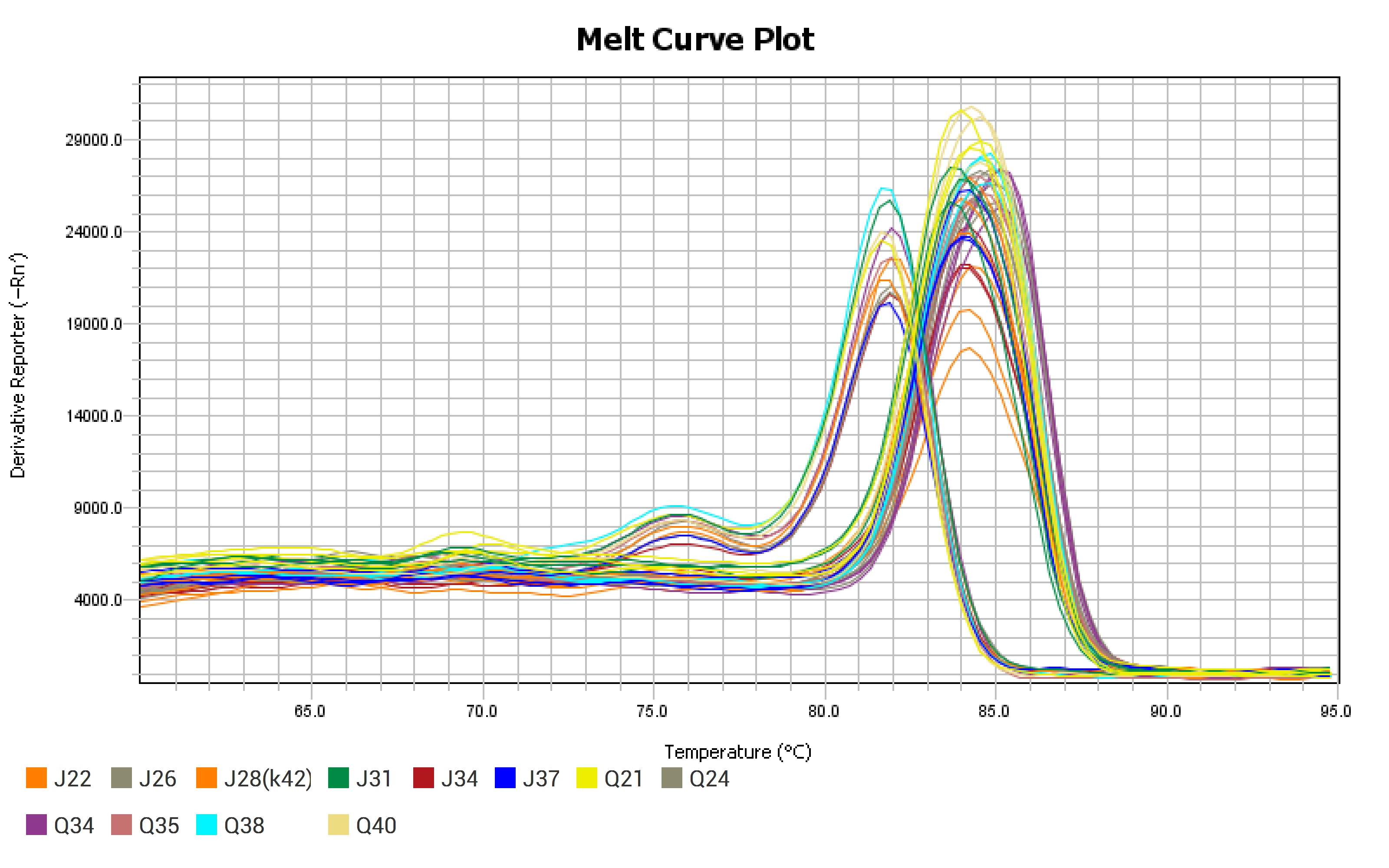

Supplement: Supplementary file 2 [file Data_Sheet_2.zip › data 2/SLC31A1-Melt Curve Plot.jpg]
